# A Transmembrane Protein WAI‐B2 Confers Multiple Disease Resistance in Wheat by Activating Autoimmunity

**DOI:** 10.1002/advs.202511576

**Published:** 2025-10-28

**Authors:** Wenling Li, Yongxing Chen, Lei Dong, Guanghao Guo, Huaizhi Zhang, Tao Shen, Gaojie Wang, Lingli Dong, Ping Lu, Miaomiao Li, Dan Qiu, Keyu Zhu, Beibei Li, Yikun Hou, Xuejia Cui, Baoge Huang, Fugang Yang, Hongkui Fu, Delin Li, Zhan Li, Jinghuang Hu, Yunfeng Qu, Huanhuan Lou, Shisheng Chen, Zaifeng Li, Ling Kang, Wuyun Yang, Chengguo Yuan, Hongjie Li, Yusheng Zhao, Zhiyong Liu, Haiyan Jia, Qiuhong Wu

**Affiliations:** ^1^ Institute of Biotechnology Xianghu Laboratory Hangzhou 311231 China; ^2^ State Key Laboratory of Seed Innovation Institute of Genetics and Developmental Biology Chinese Academy of Sciences Beijing 100101 China; ^3^ Nanjing Agricultural University Jiangsu 210018 China; ^4^ Institute of Advanced Agricultural Sciences Peking University Weifang 261200 China; ^5^ College of Plant Protection Hebei Agricultural University Baoding 071001 China; ^6^ The Crop Research Institute Ningxia Academy of Agriculture and Forestry Science Yinchuan 750002 China; ^7^ Crop Research Institute Sichuan Academy of Agriculture Sciences Chengdu 610066 China; ^8^ Hebei Gaoyi Stock Seeds Farm Gaoyi 051330 China

**Keywords:** artificial intelligence crop design, autoimmunity, multiple disease resistance, transmembrane protein, wheat

## Abstract

Wheat (*Triticum aestivum* L.) is one of the world's most important food crops and its production is frequently threatened by diseases caused by several biotrophic pathogens, including stripe rust, leaf rust, stem rust and powdery mildew. Identifying and cloning genes that confer resistance to multiple‐diseases hold significant value for breeding wheat cultivars with broad‐spectrum disease resistance. In this study, a *wheat autoimmunity*‐*B2* (*WAI*‐*B2*) gene is cloned from an ethyl methanesulfonate (EMS)‐induced wheat autoimmunity mutant, 8P4087, and its role in resistance against multiple foliar diseases is characterized. *WAI‐B2* encodes a unique transmembrane protein that provides resistance to powdery mildew, stripe rust, leaf rust and stem rust in wheat. Further analysis reveals that WAI‐B2 interacts with the TaHsp90 and TaHsp70, which is crucial for cell stabilization, signal transduction and programed cell death (PCD). Used AlphaFold 2 and SWISS‐MODEL to predict the optimal amino acid substitution and hydrogen bond interaction sites, a series of new *WAI‐B2* alleles is designed, and alleles capable of causing mild cell death in *N*. *benthamiana* are obtained. This study provides valuable insights into the potential of artificial intelligence (AI)‐assisted approaches for designing disease‐resistant crops in the future.

## Graphical abstract

Wheat production is under threat from multiple diseases. This study clones the wheat autoimmunity gene *WAI‐B2* from an EMS‐induced mutant that encodes a distinct transmembrane protein conferring resistance to wheat rusts and powdery mildew ‐ two major diseases affecting wheat crops. Additionally, artificial intelligence designed novel *WAI‐B2* alleles provides valuable insights for breeding advanced disease‐resistant crops.

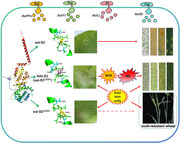

## 1 Introduction

Crop diseases pose a significant threat to agricultural productivity and quality. Developing wheat cultivars with high yield and robust disease resistance remains a key objective in wheat breeding programs.^[^ ^1^, ^2^ ^]^ Most disease resistance (*R*) genes in cereal crops, including wheat, belong to the intracellular nucleotide‐binding leucine‐rich repeat (NLR) receptor superfamily. These genes are highly polymorphic and typically provide resistance to specific pathogen isolates. However, cultivars harboring race‐specific *R* genes often face directional selection effects during large‐scale breeding and cultivation, which can lead to the eventual loss of disease resistance.^[^ ^3^ ^]^ Achieving durable disease resistance is a critical goal in crop improvement. One promising approach to mitigate the loss of resistance associated with single *R* genes is the simultaneous introduction of multiple *R* genes, enabling the development of cultivars with broad‐spectrum, multi‐disease and long‐lasting resistance.^[^ ^4^ ^]^

Wheat Autoimmunity (WAI) mutants exhibit constitutively activated defense responses even in the absence of pathogen infection. These mutants are characterized by hypersensitive responses (HR) with histological and cytological features resembling typical disease resistance reactions, such as bursts of reactive oxygen species (ROS) and the upregulation of pathogenesis‐related (PR) genes. WAI proteins are typically associated with processes like cell development, apoptosis, stress tolerance, and disease resistance, making them valuable resources for breeding disease‐resistant crops. As such, they hold great potential for developing cultivars with broad‐spectrum, multi‐disease resistance.^[^ ^5^ ^]^ Autoimmunity is often referred to as “lesion mimic”, a phenomenon where symptoms resembling leaf spot disease occur spontaneously, independent of pathogen presence. To date, several lesion mimic mutants (LMMs) have been identified in wheat, many of which exhibit enhanced disease resistance.^[^ ^6^, ^7^, ^8^, ^9^, ^10^, ^11^, ^12^ ^]^ However, to date, only one *LMM* gene has been cloned, *lm34* encodes a typical CC‐NB‐LRR protein, which can enhance powdery mildew resistance.^[^ ^13^ ^]^ Understanding of the role WAI mutants play in wheat disease resistance is still limited.

The leaf tip necrosis mutant represents another type of autoimmunity mutant with pleiotropic effects that confer resistance to multiple diseases. For example, pleiotropic loci such as *Lr34*/*Yr18*/*Pm38*/*Sr57*/*Sb1*/*Ltn1* ^[^ ^14^, ^15^ ^]^ and *Lr67*/*Yr46*/*Pm46*/*Sr55*/*Ltn3*,^[^ ^16^, ^17^, ^18^ ^]^ which encode transmembrane proteins, activate immune responses by accumulating substrates during transmembrane transport. While these plants often display undesirable leaf tip necrosis traits, understanding the genetic basis of autoimmunity mutant phenotypes can provide valuable insights for achieving high grain yields by deciphering their roles in disease resistance, environmental stress responses, and photosynthesis efficiency.^[^ ^19^ ^]^ Therefore, cloning *WAI* genes and elucidating their functions are essential for uncovering the pathways involved in PCD, disease resistance mechanisms, and their potential applications in wheat disease resistance breeding.

Molecular chaperones, such as Heat Shock Protein 70 (Hsp70) and Heat Shock Protein 90 (Hsp90), play critical roles in assisting protein folding and remodeling client proteins, processes essential for managing cellular stress.^[^ ^20^ ^]^ Due to its fundamental role in cellular function, Hsp90 is intricately linked to various forms of PCD and is implicated in numerous diseases.^[^ ^21^ ^]^ Notably, some plant NLR proteins have been shown to interact with both Hsp90 and SGT1 (suppressor of the G2 allele of *skp1*).^[^ ^22^ ^]^ Furthermore, Hsp90, along with SGT1 and RAR1 (required for Mla12 resistance), has been demonstrated to contribute to both basal immunity and R protein‐mediated resistance in plants.^[^ ^23^ ^]^ In contrast to Hsp90, Hsp70 plays a significant role in regulating the replication and movement of pathogenic bacteria within cells. Elevated expression of Hsp70 has been found to enhance bacterial infection.^[^ ^24^ ^]^ Together, Hsp90 and Hsp70 act as critical regulatory nodes in pathways such as apoptosis, autophagy, necroptosis, ferroptosis, and other forms of PCD.

In this study, we report the characterization, map‐based cloning, and functional validation of a wheat autoimmunity gene, *WAI‐B2*, derived from EMS induced WAI mutant 8P4087 from winter wheat cultivar Nongda 399 (ND399). *WAI‐B2* encodes a transmembrane protein of unknown function and exhibits enhanced resistance to stripe rust, leaf rust, stem rust and powdery mildew. WAI‐B2 interacts with TaHsp90 and TaHsp70, and a site mutation at the 425th amino acid residue of WAI‐B2 has been identified as critical for the activation of autoimmunity.

## 2 Results

### 2.1 Phenotypic Characterization of WAI Mutant 8P4087

**Figure 1:**
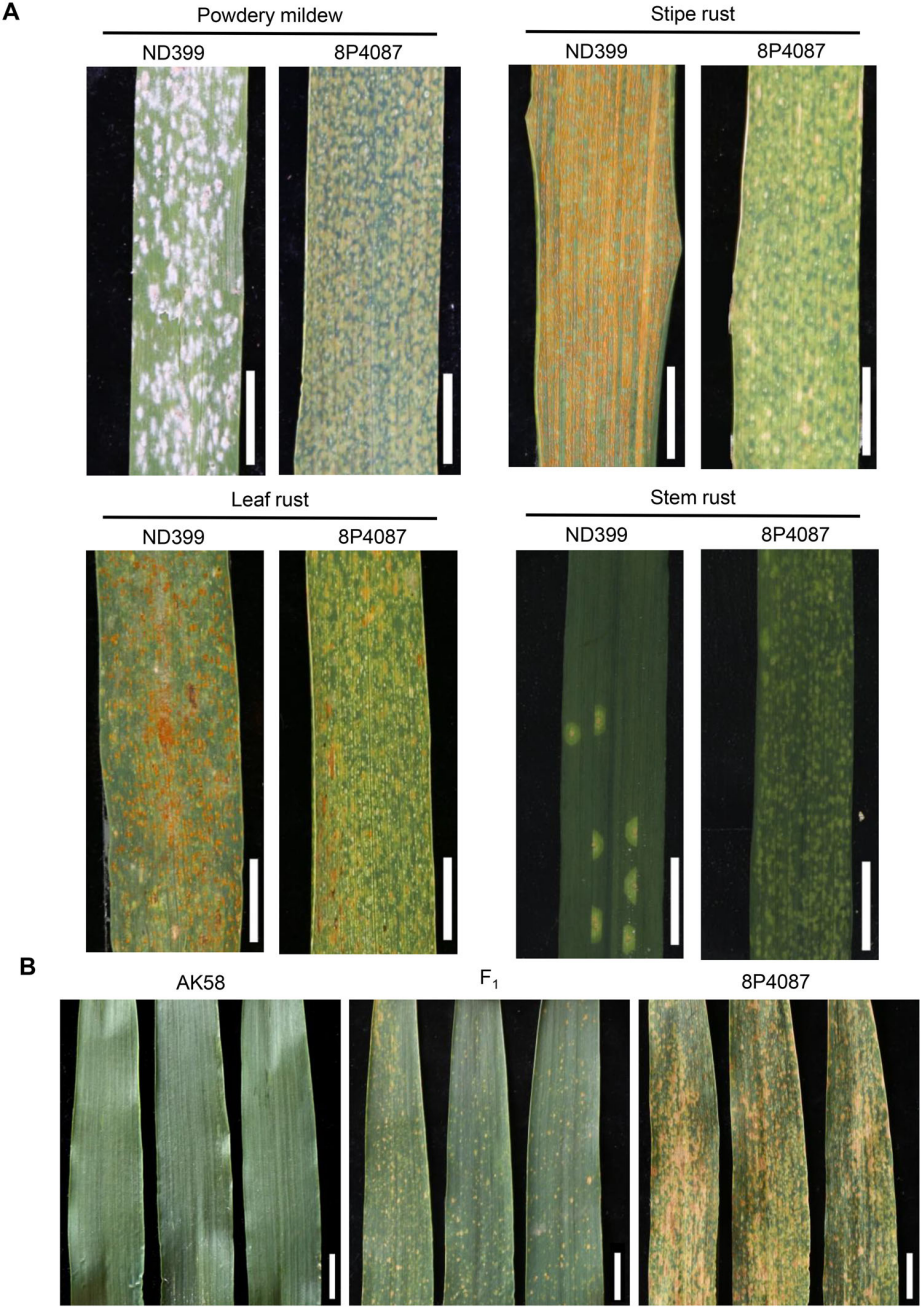
Disease resistance assessments of ND399 and WAI mutant 8P4087, and leaf spot phenotypes of AK58, 8P4087 and their F_1_ hybrid. A) Reactions of wild‐type (WT) ND399 (left) and 8P4087 (right) to powdery mildew, stripe rust, leaf rust and stem rust at the adult‐plant stage. B) Leaf spot morphologies of parental wheat line AK58 (left), WAI mutant 8P4087 (right) and their F_1_ hybrids (medium). Scale bars: 1 cm.

The WAI mutant 8P4087, identified from an EMS‐induced mutation population of the winter wheat cultivar ND399, exhibited spontaneous leaf spots formation. The necrosis phenotype in 8P4087 began in the primary leaf at the three‐leaf‐stage and progressively spread across the leaves, in contrast to the wild‐type (WT) ND399 (Figure S1A, Supporting Information). 3,3'‐diaminobenzidine (DAB) staining revealed dark brown‐red coloration in 8P4087 leaves, indicating pronounced accumulation of H_2_O_2_, compared to the WT (Figure S1B, Supporting Information). Additionally, trypan blue staining showed noticeable changes in cell membrane permeability in 8P4087 leaves, accompanied by HR, PCD, and excessive accumulation of ROS (Figure S1B, Supporting Information). Cultivation of 8P4087 in Murashige and Skoog (MS) medium under sterile conditions at 16°C still resulted in spontaneous spot formation in young leaves (Figure S2, Supporting Information). Compared to the WT, 8P4087 exhibited reduced plant height (PH), grain number per spike (GNS), thousand‐grain weight (TGW), and grain number per plant, but no significant difference in spike length, spikelet number, flag leaf length, or flag leaf width (Figure S3, Supporting Information). Interestingly, 8P4087 displayed resistance to powdery mildew, stripe rust, and leaf rust at the adult‐plant stage under field conditions with artificial inoculation (**Figure** **1**A). However, both ND399 and 8P4087 exhibited high resistance to stem rust.

### 2.2 Genetic Analysis and Map‐Based Cloning

**Figure 2:**
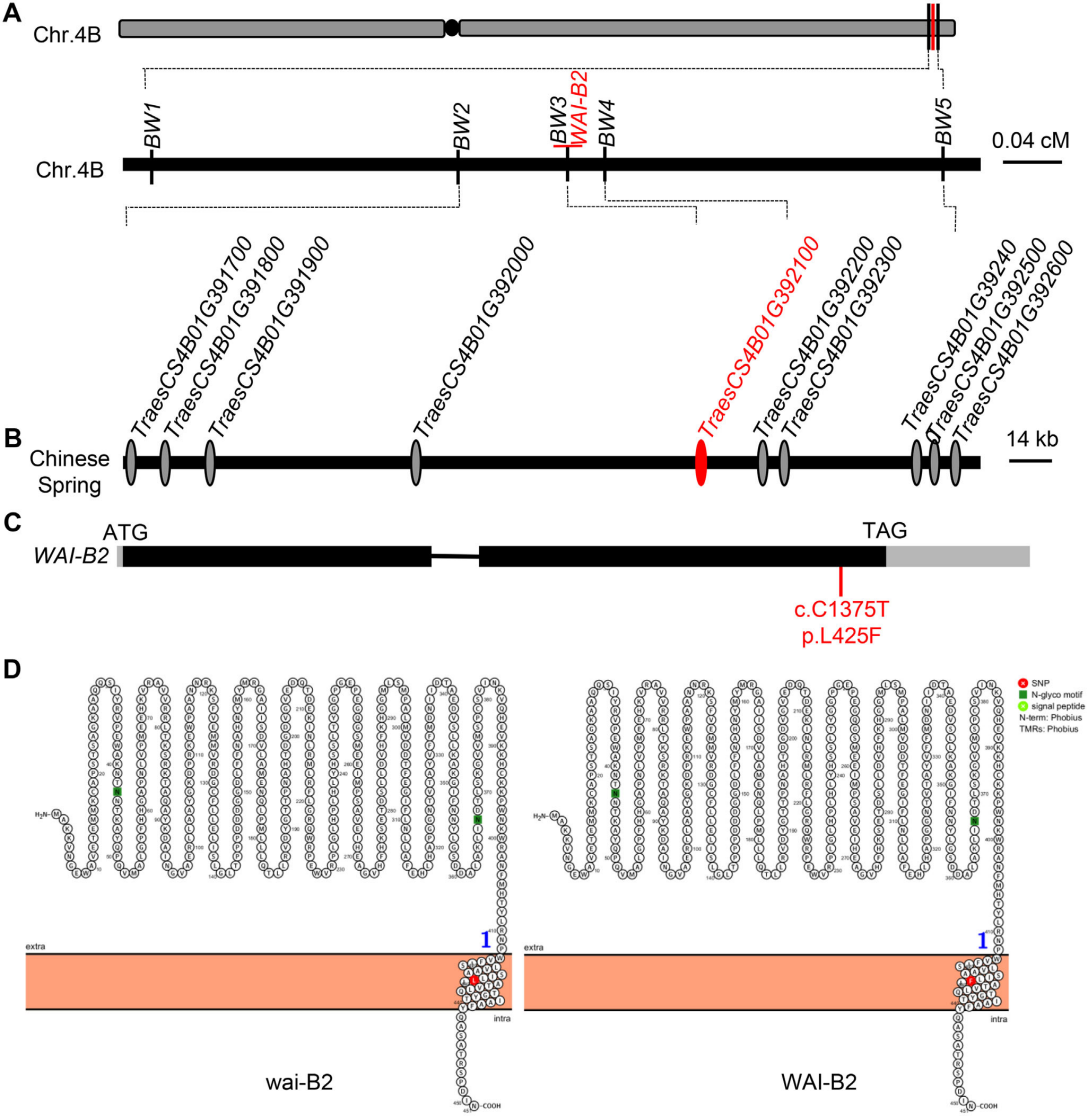
Map‐based cloning of *WAI*‐*B2*. A) Genetic linkage map of *WAI*‐*B2* generated using 3054 F_2_ plants derived from 8P4087 × AK58 and 8P4087 × KN9204 populations. B) Predicted genes within the *WAI‐B2* mapping interval in Chinese Spring. C) Gene structure and allelic differences between *WAI‐B2* and *wai*‐*B2*. Black box indicates the exons. The red bar indicates the C/T mutation site in cDNA (C) that changes amino acid (P) from leucine (Leu, L) to phenylalanine (Phe, F) at the 425th residue. D) *WAI‐B2* encodes a transmembrane protein of 451 amino acids (aa) and the L to F substitution was located in the predicted transmembrane domain.

8P4087 was crossed with a widely cultivated common wheat variety Aikang 58 (AK58) to generate a segregating population for genetic analysis. The F_1_ plant exhibited an intermediate phenotype (Figure 1B), suggesting the semi‐dominance nature of the mutation. Genetic analysis of the F_2_ progenies revealed that the spontaneous spot phenotype was governed by a single incomplete dominant gene, designated *WAI‐B2* (Table S1, Supporting Information). BSR‐Seq analysis pinpointed the mutation locus to the long arm of chromosome 4B (Table S2, Supporting Information). Using a mapping population comprising 809 F_2_ plants of the cross 8P4087 × AK58, *WAI‐B2* was positioned within a 1.17 cM genetic interval between the SNP marker *BW2* and InDel marker *BW5* (Table S3, Supporting Information), corresponding to a 205 kb genomic region in the Chinese Spring RefSeq v1.0 genome http://wheatomics.sdau.edu.cn/), containing eight putative protein coding genes (**Figure** **2**A,B; Table S4, Supporting Information). To further narrow down the physical mapping interval, another population consisting of 2,245 F_2_ plants derived from the cross 8P4087 × KN9204 was genotyped and phenotyped (Table S1, Supporting Information). Eventually, *WAI‐B2* was mapped in a 0.11 cM genetic interval between SNP markers *BW2* and *BW4*, corresponding to a 167 kb genomic region in the Chinese Spring RefSeq v1.0, which contains five putative genes (Figure 2A,B). Sequence comparisons of the five genes between ND399 and 8P4087 revealed a single nucleotide substitution (C to T) at the 2nd exon of *TraesCS4B01G392100*, resulting in an amino acid mutation from leucine (Leu) to phenylalanine (Phe) at the 425th residue (Figure 2C). This mutation occurs within the predicted transmembrane domain of the encoded protein (Figure 2D). The *WAI‐B2* candidate allele, corresponding to *TraesCS4B01G392100*, is exclusively located on wheat chromosome 4B, with no homoeolog gene detected on chromosomes 4A or 4D of Chinese Spring and other available wheat genome assemblies.

Quantitative reverse transcription PCR (qRT‐PCR) analysis showed that the expression levels of both *WAI‐B2* in 8P4087 and WT allele *wai‐B2* in ND399 were extremely low prior to the appearance of the leaf spot phenotype. However, once the leaf spot phenotype was observed in 8P4087, the expression level of *WAI‐B2* was significantly higher than that of *wai‐B2*. Similarly, qRT‐PCR analysis revealed comparable trends of *TaPR1* expression. These findings demonstrated that in addition to the presence of mutation sites, autoimmunity phenotype in 8P4087 also depended on the upregulation of *WAI‐B2* (Figure S4, Supporting Information).

### 2.3 Function Validation of *WAI‐B2* by Transgenic Complementation

**Figure 3:**
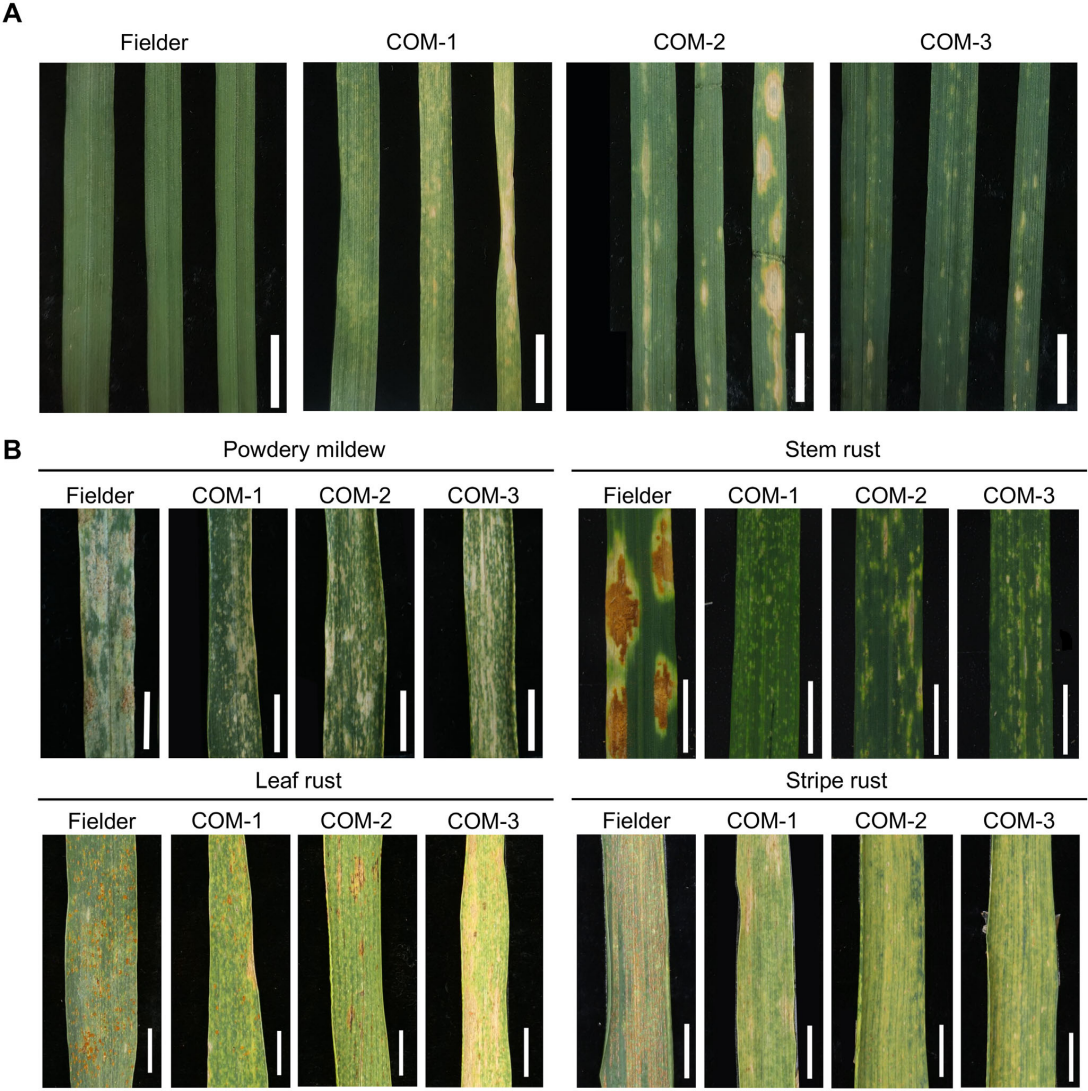
Transgenic validation of *WAI‐B2*. A) T_1_ plants transformed with *WAI‐B2* displayed spontaneous spots phenotype. COM‐1, COM‐2, and COM‐3 are the *WAI‐B2* transgenic positive plants, and Fielder is the control. B) Reactions of Fielder and *WAI‐B2* transgenic lines to powdery mildew, stem rust, leaf rust, and stripe rust at the adult plant stage. Scale bars: 1 cm.

To evaluate whether the mutated *WAI‐B2* allele in 8P4087 is responsible for the spontaneous leaf spot phenotype, we constructed a complementation plasmid containing the genomic sequence of the *WAI‐B2*, flanked by a 2,473 bp upstream promoter region and a 1,260 bp downstream sequence (Table S3, Supporting Information). Using *Agrobacterium‐*mediated transformation, we introduced the plasmid into the wheat variety Fielder and obtained three transgenic lines. All three positive lines harboring the transgene displayed the spontaneous leaf spot phenotype, confirming successful complementation of the phenotype, whereas the non‐transgenic Fielder has a normal phenotype (**Figure** **3**A). qRT‐PCR analysis showed that the expression levels of *WAI‐B2* in the transgenic lines were extremely low prior to the appearance of the leaf spot phenotype, although significantly higher than the expression level of *wai‐B2* allele in Fielder. However, after the leaf spot phenotype appeared, the expression levels of *WAI‐B2* were significantly higher in the transgenic lines. Similarly, qRT‐PCR analysis revealed comparable trends of *TaPR1* expression in the transgenic lines (Figure S5, Supporting Information). According to the assessment of disease resistance, the transgenic line COM‐3 carrying *WAI‐B2* was resistant to powdery mildew and stem rust at the seedling stage (Figure S6, Supporting Information). In addition, to investigate whether *WAI‐B2* can confer multiple disease resistance at adult plant stage, three transgenic lines carrying *WAI‐B2* were evaluated for resistance to powdery mildew, stem rust, leaf rust, and stripe rust at the adult plant stage, and the results all showed enhanced disease resistance, suggesting that *WAI‐B2* provided all‐stage multiple disease resistance (Figure 3B).

**Figure 4:**
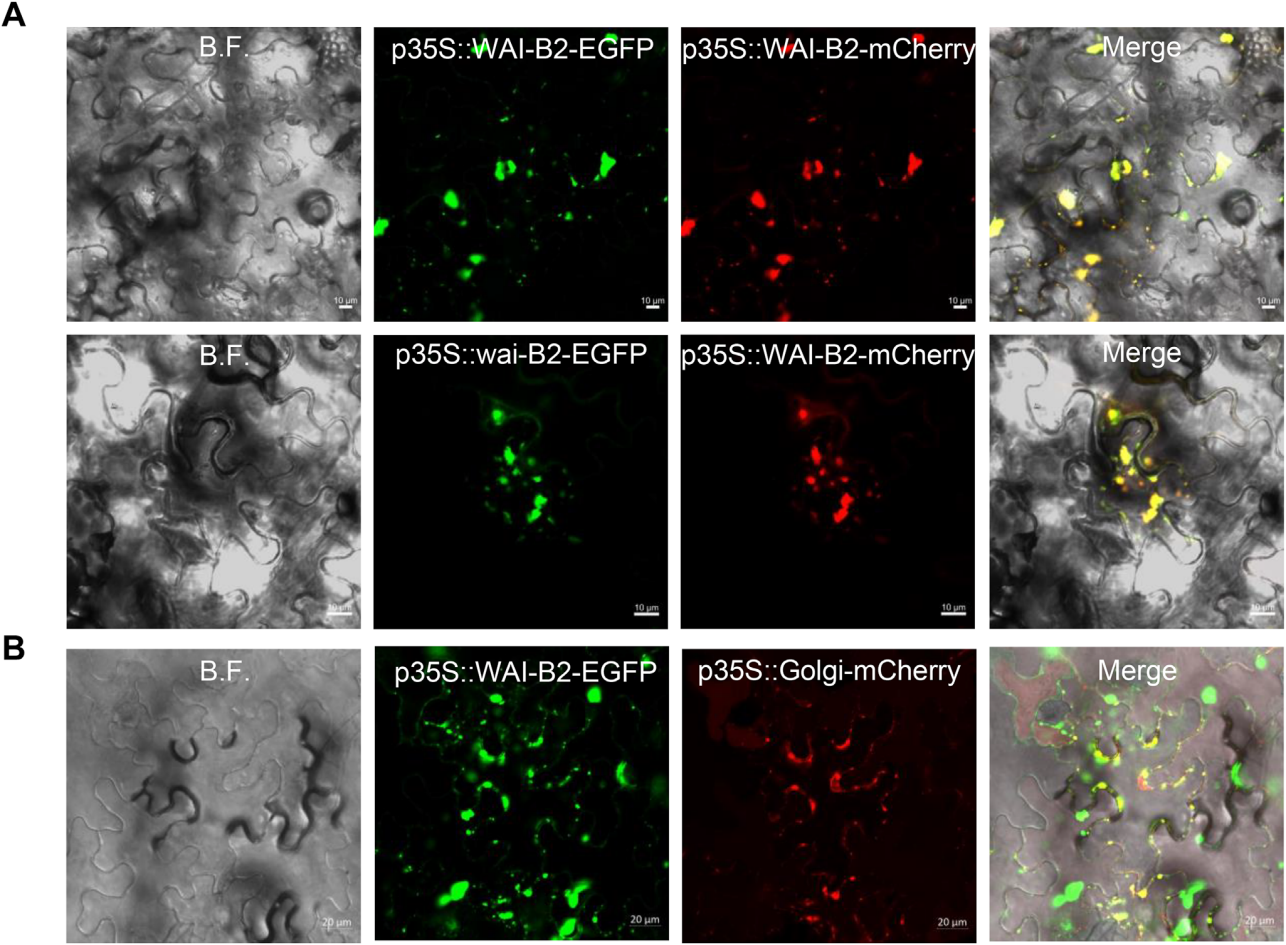
The subcellular localizations of WAI‐B2. A) Co‐localization of p35S::WAI‐B2‐mCherry with p35S::wai‐B2‐EGFP in *N*. *benthamiana*. p35S::WAI‐B2‐mCherry was transiently co‐expressed with p35S::WAI‐B2‐EGFP (top) as control. Scale bars: 10 µm. B) Co‐localization of p35S::wai‐B2‐EGFP and Golgi marker (p35S::Golgi‐mCherry) in *N*. *benthamiana*. Scale bars: 20 µm.

The subcellular localizations of WAI‐B2 and wai‐B2 proteins were analyzed by fusing them separately with enhanced green fluorescent protein (EGFP) and co‐expressing them with p35S::WAI‐B2‐mCherry in *Nicotiana benthamiana* (*N*. *benthamiana*) leaves via agroinfiltration. Confocal laser scanning microscopy images revealed that the green fluorescence of p35S::WAI‐B2‐EGFP and p35S::wai‐B2‐EGFP co‐localized with the red fluorescence of p35S::WAI‐B2‐mCherry, indicating that the Leu to Phe mutation does not alter the subcellular localization of the protein. Moreover, fluorescence signals exhibited a punctate pattern in the cytoplasm and were targeted to Golgi apparatus (**Figure** **4**A). In order to further analyze the effects of transmembrane regions on subcellular localization, the WAI‐B2 was divided into two parts, the N‐terminus from 1‐440 aa and the C‐terminus from 413–451 aa, according to the transmembrane domain. Both parts included the transmembrane region from 413–440 aa of WAI‐B2. The two sequences were inserted downstream of the 35S promoter in the pCAMBIA1300 vector and fused with EGFP or mCherry and co‐expressed in *N*. *benthamiana* leaves via agroinfiltration. Subcellular localization results showed that p35S::WAI‐B2‐N‐mCherry was co‐localized with p35S::WAI‐B2‐EGFP. However, p35S::WAI‐B2‐C‐mCherry was not only co‐localized with p35S::WAI‐B2‐EGFP but also co‐localized with the plasma membrane and nuclear membrane (Figure S7A, Supporting Information). Moreover, fluorescence signals exhibited a punctate pattern in the cytoplasm and were targeted to Golgi apparatus (Figure 4B). To further investigate subcellular localization, vector WAI‐B2‐163UBI‐mCherry was transfected into wheat leaf mesophyll protoplasts along with WAI‐B2‐163UBI‐EGFP and wai‐B2‐163UBI‐EGFP. The majority of green fluorescence signals appeared fragmented but also showed co‐localization in some protoplasts, and were targeted to Golgi apparatus (Figure S7B, Supporting Information). Based on the prediction that WAI‐B2 functions as a transmembrane protein, it is likely involved in signaling and transport processes during stress responses and PCD.

### 2.4 WAI‐B2 Interacts with TaHsp90 and TaHsp70

**Figure 5:**
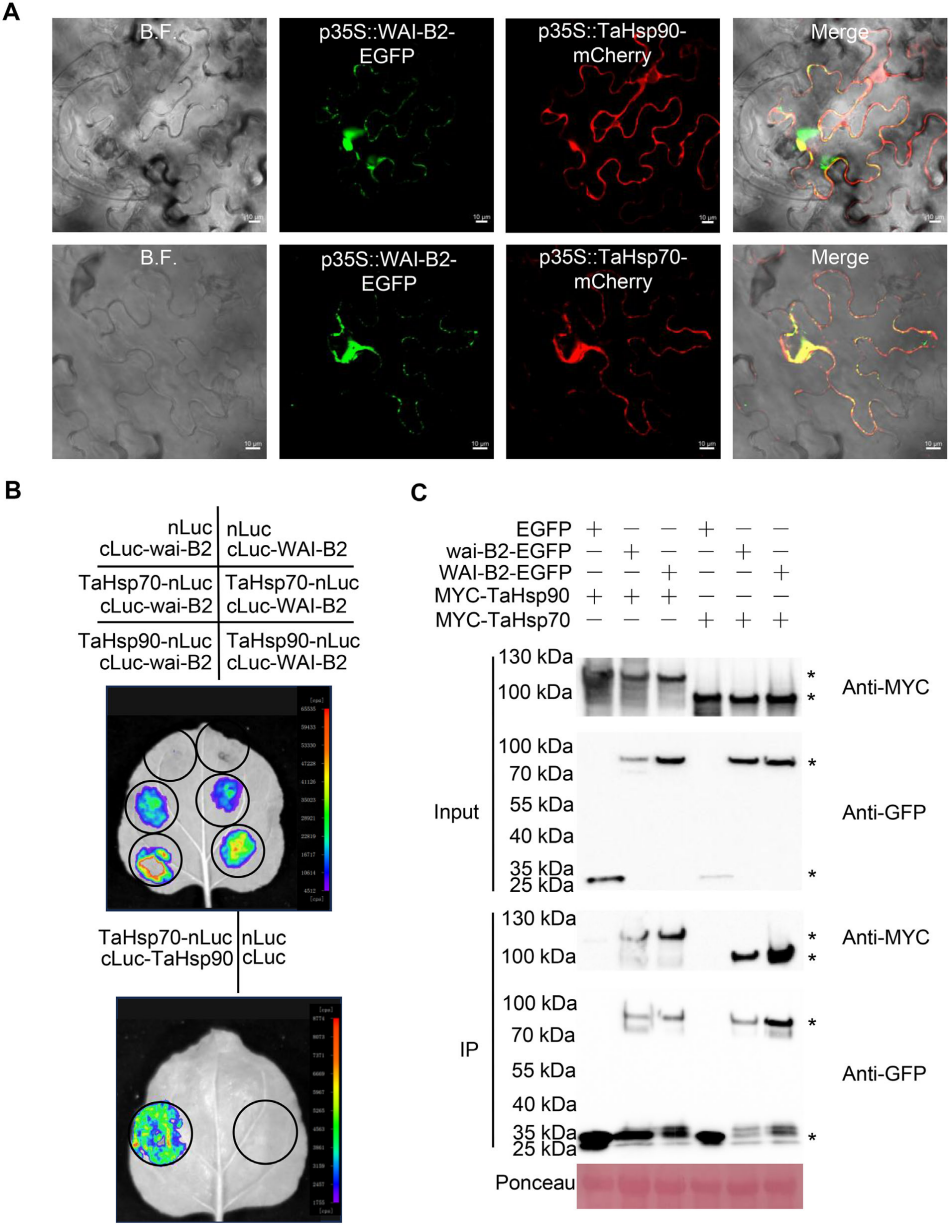
WAI‐B2 interacts with the TaHsp90 and TaHsp70. A) p35S::WAI‐B2‐EGFP was transiently co‐expressed with a red fluorescent protein‐tagged with TaHsp90 and TaHsp70 marker in *N*. *benthamiana* leaves. Scale bars: 10 µm. B) LCI assay of cLuc‐wai‐B2 and cLuc‐WAI‐B2 with TaHsp90‐nLuc and TaHsp70‐nLuc in *N*. *benthamiana* leaves. The pseudo‐color bar indicates the range of luminescence intensity. C) Confirmation of the interaction between wai‐B2 and WAI‐B2 with TaHsp90 and TaHsp70 by Co‐IP assays. Western blots of total proteins transiently expressing the marked constructs and proteins eluted from GFP beads were detected using the anti‐GFP or anti‐Myc antibody. * indicates the position of the target fusion proteins.

To further explore the mechanism underlying the WAI‐B2‐induced HR‐like reaction, we conducted immunoprecipitation followed by mass spectrometry (IP‐MS) to delineate the WAI‐B2 interactome. Using WAI‐B2‐pS1300‐EGFP and wai‐B2‐pS1300‐EGFP vectors expressed in *N*. *benthamiana* leaves, we identified two heat shock proteins, NbHsp90 and NbHsp70, as interacting proteins of WAI‐B2. Using the sequences of homologous genes *TraesCS5D01G268000* and *TraesCS3D01G352400* in the Chinese spring reference genome as templates, primers were designed and used to amplify *TaHsp90* and *TaHsp70* from 8P4087 (Tables S5–S7, Supporting Information). Subsequent analyses, including subcellular localization, luciferase complementation imaging (LCI), and Co‐immunoprecipitation (Co‐IP) assays, confirmed the interactions between WAI‐B2 with the TaHsp90 and TaHsp70. Co‐expression of p35S::WAI‐B2‐EGFP with red fluorescent protein‐tagged TaHsp70 and TaHsp90 markers revealed sporadic co‐localizations of p35S::WAI‐B2‐EGFP with each component (**Figure** **5**A). LCI assays in *N*. *benthamiana* leaves further supported these interactions, as strong fluorescence signals were observed when cLuc‐WAI‐B2 and cLuc‐wai‐B2 were co‐expressed with TaHsp70‐nLuc and TaHsp90‐nLuc, respectively (Figure 5B). However, TaDnaJ, the co‐chaperones of TaHsp70 and TaHsp90, could not interact with cLuc‐WAI‐B2 or cLuc‐wai‐B2 (Figure S8A, Supporting Information). Furthermore, protein‐protein interaction between TaHsp90‐nLuc and cLuc‐TaHsp70 was also detected using LCI assays in *N*. *benthamiana* leaves (Figure 5B). In vitro Co‐IP assays demonstrated the co‐immunoprecipitation of WAI‐B2‐pS1300‐EGFP and wai‐B2‐pS1300‐EGFP with TaHsp90‐Myc and TaHsp70‐Myc (Figure 5C), respectively. In addition, qRT‐PCR assay shows *TaHsp70* and *TaHsp90* exhibit expression patterns similar to *WAI‐B2* (Figure S8B, Supporting Information). These results suggested that WAI‐B2 may play a role in innate immunity by functioning through TaHsp90 and TaHsp70.

### 2.5 Evolutionary Analysis of WAI‐B2 Homologs

Through homologous sequence alignment of wheat genomes, we identified three haplotypes of *WAI‐B2* in sequenced common wheat genome. Hap‐1 is the unique *WAI‐B2* allele showing autoimmunity phenotype. *wai‐B2* is Hap‐2 that corresponded to Fielder, Norin61, Xinong6028, and Yangmai158, etc. Hap‐3 is Chinese Spring, AK58, and KN9204, etc. In tetraploids, Durum wheat Svevo and Kronos are Hap‐3, while wild emmer Zavitan is Hap‐2 (Figure S9 and Table S8, Supporting Information).

Comparative analysis of WAI‐B2 orthologs using publicly available *Triticeae* genome data (http://wheatomics.sdau.edu.cn/) indicated that wai‐B2 present only in the 4B and 4S of common wheat, wild emmer wheat, durum wheat, and the S genome. These results suggested that wai‐B2 likely originated from the S genome (Figure S10A, Supporting Information). Homology search of the WAI‐B2 protein in *Arabidopsis thaliana*, *Zea mays* and *Oryza sativa* revealed that only Zm00001d052636 in *Zea mays* had more than 50% sequence identity with WAI‐B2. The most homologous protein in *Oryza sativa* is LOC_Os11g34090.1, which displays 41% sequence identity, while the most homologous protein in *A*. *thaliana* is AT3G50120 with 33% sequence identity. Notably, all of the homologous proteins were annotated as unknown function transmembrane protein. Phylogenetic tree analysis further demonstrated that WAI‐B2 is a distinct protein within the *Triticeae* family, showing low similarity to proteins from *A. thaliana*, *Z. mays* and *O. sativa* (Figure S10B, Supporting Information).

### 2.6 Artificial Intelligence Assisted WAI‐B2 Variants Designing

**Figure 6:**
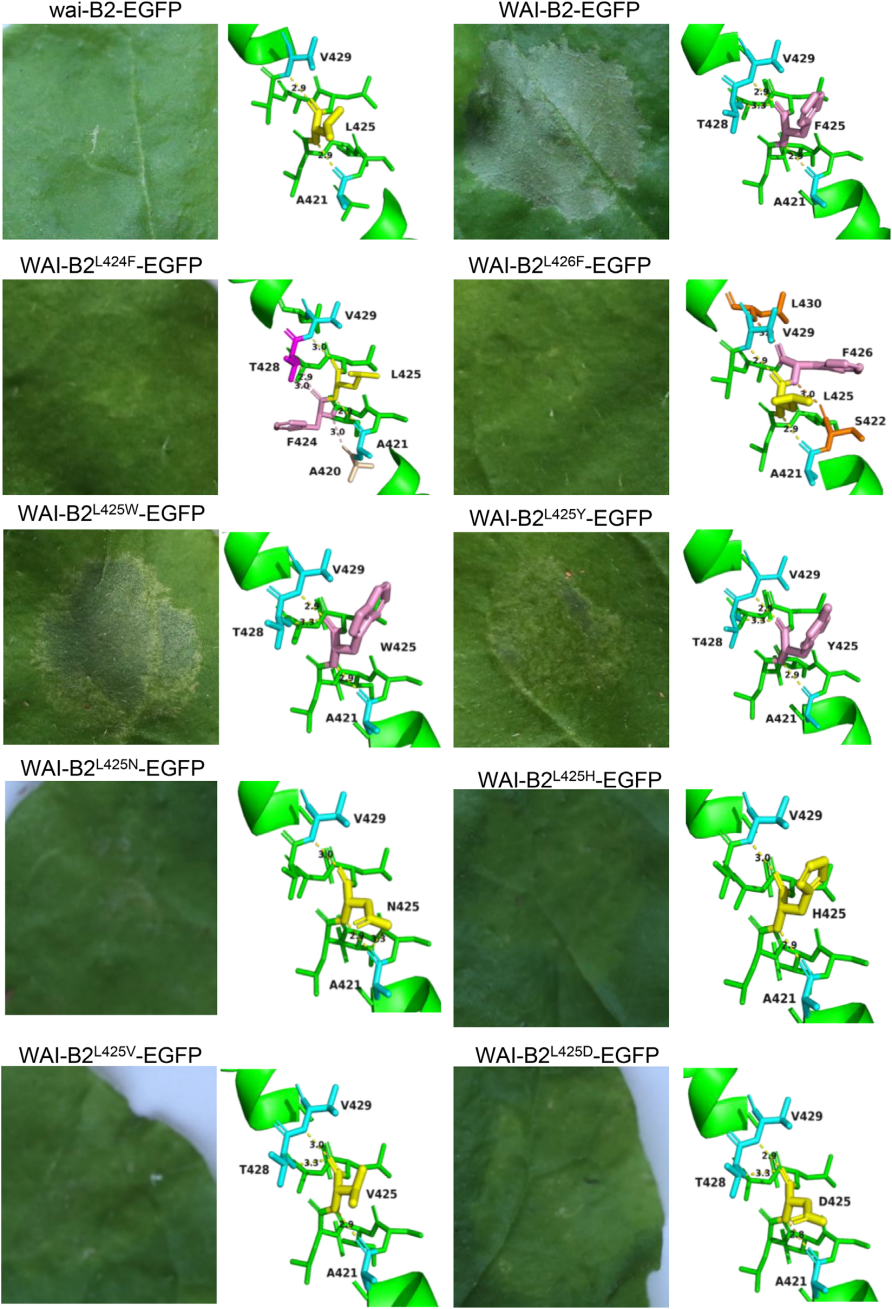
Cell‐death‐inducing activity of WAI‐B2 variants in *N*. *benthamiana*. The site mutations on the 425th residue of WAI‐B2 protein was expressed in *N*. *benthamiana* leaves by agroinfiltration. Cell death was observed at 72 hpi. The same results were obtained in three independent experiments. Structures of proteins predicted by SWISS‐MODEL and painted by PyMol. The 425th amino acid was marked in yellow, while amino acid containing benzene rings was distinctly highlighted in purple. Amino acids engaged in hydrogen bonding with the 424^th^, 425^th^, and 426th amino acids are indicated in pink and deep purple, blue, and orange, respectively. The distances between these amino acids were annotated, providing a comprehensive structural understanding of the WAI‐B2 variants.

To investigate the biological function of WAI‐B2 and assess whether the WAI‐B2‐EGFP fusion influences its function, we conducted a cell‐death assay in *N*. *benthamiana*. Overexpression of *WAI‐B2‐EGFP* induced an HR‐like reaction at 72 h post infiltration (hpi) in *N*. *benthamiana*, whereas patches infiltrated with the *wai‐B2‐EGFP* vector did not display an HR‐like phenotype (**Figure** **6**). Given that the mutation site (L425F) is surrounded by leucine (L424L425L426), we constructed site‐directed mutations at positions L424 and L426 to generate WAI‐B2^L424F^ and WAI‐B2^L426F^ variants, which were then expressed in *N*. *benthamiana* leaves. Neither of these variants induced an HR‐like phenotype, indicating that the L425 residue in the WAI‐B2 protein is a key site for its autoimmune function. To further explore the effect of other amino acid variations at position 425 on the autoimmune function of WAI‐B2 protein, we designed additional variants to alter the interacting properties at this site and tested them in *N*. *benthamiana* leaves. The results showed that both WAI‐B2^L425W^, WAI‐B2^L425Y^ and WAI‐B2^L425N^ caused cell necrosis with varying degree of HR severity, while WAI‐B2^L425D^, WAI‐B2^L425H^, and WAI‐B2^L425V^ did not induce an HR‐like reaction (Figure 6). These findings confirm the importance of the 425th amino acid residue in inducing a cell‐death‐like reaction when ectopically expressed in *N*. *benthamiana*. Furthermore, the varying degrees of cell necrosis caused by different amino acid substitutions at position 425 highlight the importance of this site. These results provide a theoretical basis for the artificial modification of WAI‐B2 by introducing novel amino acid replacement to modulate its function.

## 3 Discussion

Disease resistance is one of the most important traits in crop breeding. Most plant disease resistance genes belong to complex gene families, such as the NLR class of intracellular receptors or extracellular membrane‐anchored receptor‐like proteins (RLPs, known as RLKs when containing an intracellular kinase). However, pathogens continuously mutate and evolve, often overcoming the efficacy of single resistance genes through the emergence of new virulent strains. In contrast, species non‐specific or race non‐specific broad‐spectrum resistance genes offer long‐term, durable resistance against multiple pathogens. Here, we demonstrate that *WAI‐B2* encodes a unique transmembrane protein that triggers wheat autoimmunity, thereby conferring resistance to a variety of pathogens in wheat.

Plants exhibit autoimmunity against infection caused by multiple plant pathogens through mechanisms that involve the co‐regulation of multiple defense pathways, rather than being attributable to a single cause. Some forms of autoimmunity arise from mutations in disease‐resistance genes that alter the signaling pathways initiating HR, such as the gene *rp1* in maize.^[^ ^25^ ^]^ Other types of autoimmunity are caused by mutations in catalytic enzymes or metabolites involved in normal metabolic pathway.^[^ ^26^, ^27^ ^]^ Examples include the maize gene *les22*, which disrupts the porphyrin metabolic pathway,^[^ ^28^ ^]^ the *modl* gene in *Arabidopsis*, which impairs fatty acids biosynthesis,^[^ ^29^ ^]^ and the LSD1 mutant in *Arabidopsis*, which leads to the rapid accumulation of O^2‐^ and H_2_O_2_.^[^ ^30^ ^]^ In addition to these pathways, signaling pathways involving certain wheat transmembrane proteins have been shown to confer broad‐spectrum resistance to multiple pathogens. Gene complexes such as *Lr34*/*Yr18*/*Sr57*/*Pm38*/*Ltn1* and *Lr67*/*Yr46*/*Sr55*/*Pm46*/*Ltn3* encode transmembrane proteins that provide partial but durable resistance against leaf rust, yellow rust, stem rust, and powdery mildew. Similarly, the wheat aquaporin TaPIP210 activates the immune response by accumulating H_2_O_2_, enhancing resistance to powdery mildew and *Fusarium* head blight.^[^ ^31^ ^]^ Therefore, site mutations in transmembrane proteins may lead to the accumulation of substrates, causing osmotic pressure imbalance that activate immune response. In our study, we observed that overexpression of the *WAI‐B2* gene caused wheat protoplast rupture and necrosis in *N*. *benthamiana*. Furthermore, several other variants at the 425^th^ amino acid position in the transmembrane region of the WAI‐B2 triggered immune response in *N*. *benthamiana*, resulting in necrosis. These findings suggest that a site mutation in WAI‐B2 likely causes an osmotic pressure imbalance, disrupting normal PCD progression and activating immune response. Further exploration of its functions may uncover additional mechanisms underlying spontaneous immune responses and active defense signaling pathways.

Recent advancements in multifaceted methodologies combining various techniques and model systems have elucidated critical structural and mechanistic aspects of the Hsp90 and Hsp70 machinery. Hsp90 and Hsp70 have been shown to play key roles in signal transduction, cell cycle control, and transcriptional regulation. Components such as Hsp90, Hsp70, Hsp40, SGT1, and RAR1 are integral to these complexes and are involved in the regulation of *R* gene‐mediated resistance.^[^ ^32^ ^]^ In plants, Hsp90 has been found to participate in various programmed cell death (PCD) processes. As a key regulatory node in apoptosis, autophagy, and necroptosis, Hsp90 is associated with the development of numerous diseases. In plants, Hsp90 has been shown to activate NLR proteins. Together with auxiliary chaperones RAR1 and SGT1, Hsp90 regulates cytoplasmic R proteins through direct interaction with R proteins, such as RPS2 and RPS4 mentioned in the “bait model”.^[^ ^33^, ^34^ ^]^ Individually inhibiting Hsp90, RAR1, or SGT1 results in reduced plant disease resistance.^[^ ^23^, ^35^ ^]^ In rice, chitin receptor OsCERK1 interacts with Hsp90 and its chaperone Hop/Sti1 in the endoplasmic reticulum, facilitating its translocation from the endoplasmic reticulum to the plasma membrane for PAMP recognition, a process dependent on the GTPase Sar1.^[^ ^36^ ^]^ Hsp90 plays roles in both innate immune processes (PTI) and effector immune responses (ETI). Unlike Hsp70, which significantly enhances pathogen infection by regulating bacterial replication and motility, high expression of Hsp70 in cells actually weakens pathogen invasion. For example, Hsp70 is involved in the positive regulation of viral replication in tomato. When cucumber is infected with cucumber necrosis virus, Hsp70 helps the capsid protein of cucumber necrosis virus to target the chloroplast.^[^ ^24^ ^]^ In wheat, the *HSP90.2* knockout mutant in durum wheat was found to be susceptible to powdery mildew, whereas the *HSP90.2* overexpression line exhibited resistance.^[^ ^37^ ^]^ Similarly, silencing *TaHSP90.2* and *TaHSP90.3* compromised stripe rust resistance in common wheat cultivar Suwon 11.^[^ ^38^ ^]^ Furthermore, TaRAR1 and TaSGT1 has been identified as interacting partners of TaHsp90, contributing to seedling growth and stripe rust resistance in bread wheat.^[^ ^39^ ^]^ Our findings reveal that expression of *WAI‐B2* influence the expression levels of components in the TaHsp70 and TaHsp90, highlighting the importance of WAI‐B2 as a regulator of PCD and disease resistance.

Autoimmunity, activated by the plant immune system to resist various biotrophic pathogens, can significantly impact plant growth and development. For example, the rice *rbl1* mutant exhibits broad‐spectrum resistance to both *Magnaporthe oryzae* and the bacterial pathogen *Xanthomonas oryzae*, but this resistance comes at the cost of reduced yield. Using a multiplex genome editing strategy, researchers successfully developed superior alleles that preserve immune function without compromising yield. The resulting deletion mutant, *RBL1* ^Δ12^, exhibited slight necrosis during the gestation but retained blast resistance without yield loss.^[^ ^40^ ^]^ These findings underscore the effectiveness of fine‐tuning disease resistance genes to balance immunity and yield, paving the way for breeding new disease‐resistant varieties with broad‐spectrum resistance. In wheat, the L425F site mutation in the WAI‐B2 provides resistance to multiple biotrophic pathogens, but also triggers a strong HR that caused wheat leaf necrosis. By optimizing amino acid replacements at the 425^th^ position of the WAI‐B2 protein, a weaker allele, WAI‐B2^L425N^, was identified. This variant induced only mild necrosis in *N*. *benthamiana* leaves. Based on this finding, prime editing technology can effectively achieve single‐base substitutions in the *WAI‐B2* gene, thereby enabling the development of novel alleles with broad‐spectrum resistance and no significant yield penalty. This approach offers a promising strategy to achieve a balance between yield and disease resistance in wheat.

## 4 Experimental Section

### Plant Materials and Growth Conditions

WAI mutant 8P4087 was identified from an EMS‐induced mutation population of winter wheat cultivar ND399. Wheat lines and genetic mapping population were grown in Gaoyi, Hebei province, China (37°61′N, 114°61′E) during the growing seasons of 2019‐2022. A total of 809 and 2,245 F_2_ plants and their F_3_ progenies derived from the crosses of 8P4087 × Aikang 58 (AK58) and 8P4087 × Kenong 9204 (KN9204), respectively, were used for fine mapping of *WAI‐B2*. Twenty plants of 8P4087 and ND399 were randomly selected to measure the agronomic traits, including plant height, flag leaf length, flag leaf width, spikelet numbers per spike, spike length, grain number per spike, thousand‐grain weight, and grain number per plant. Disease resistance of ND399 and mutant 8P4087 were assessed in Pixian, Sichuan province (26°03′N, 97°21′E; China) for stripe rust resistance; Baoding, Hebei province (38°86′N, 115°48′E; China) for leaf rust resistance; Yinchuan, Ningxia Hui Autonomous Region (38°28′N, 106°11′E; China) for powdery mildew resistance; and Weifang, Shandong province (36°31′N, 119°25′E; China) for stem rust resistance, during the 2022‐2023 wheat growing season.

Fielder and one *WAI‐B2* transgenic line COM‐3 were planted in the greenhouse until seedling stage for the assessment of disease resistance. *Blumeria graminis* f. sp. *tritici* (*Bgt*) isolates *Bgt1* were used for identification of powdery mildew resistance;^[^ ^41^ ^]^ *Puccinia graminis* f. sp. *tritici* (*Pgt*) 34C3RTGQM was used for identification of stem rust resistance.^[^ ^42^ ^]^

Fielder and three *WAI‐B2* transgenic lines were planted in the greenhouse until adult plant stage for the assessment of disease resistance. Mixed pathogen isolates with equal amounts are used for inoculation as described previously. *Blumeria graminis* f. sp. *tritici* (*Bgt*) isolates E09, E21, HB‐24, 3–53, 5–83 were used for identification of powdery mildew resistance;^[^ ^43^ ^]^ *Puccinia triticina* (*Pt*) THJS, PGTS, THND and PHTT pathotypes were used for identification of leaf rust resistance;^[^ ^44^ ^]^ *Puccinia striiformis* f. sp. *tritici* (*Pst*) CYR32, CYR33, CYR34, ZS and Gui22‐1 races were used for identification of stripe rust resistance;^[^ ^45^ ^]^ *Puccinia graminis* f. sp. *tritici* (*Pgt*) 34C3RTGQM was used for identification of stem rust resistance.^[^ ^42^ ^]^ The phenotypic identification of disease severity was carried out by the spores or lesions occupying the surface area of flag leaf leaves at the adult plant stage. For gene expression analysis, Fielder, and three *WAI*‐*B2* transgenic lines were grown in a growth chamber set at 16°C under 16 h light/8 h dark conditions.

### Detection of H_2_O_2_ Reaction and Plant Cell Death

To detect the accumulation of H_2_O_2_, primary leaves were cut from plants of ND399 and 8P4087 at 18 d post germination (DPG) and were infiltrated in a 3,3′‐diaminobenzidine (DAB) solution (1 mg mL^−1^, pH 5.8) for 30 min under the vacuum conditions, stored in a 37°C incubator overnight, and then decolorized with absolute ethanol for 48 h. Before assessing H_2_O_2_ accumulation, leaves were washed with water and observed under the microscope. To detect PCD, primary leaves from plants of ND399 and 8P4087 were sampled at 18 DPG and were infiltrated for 10 min in boiling water with a 0.3% trypan blue solution, which was diluted (1:1, v/v) in absolute ethanol before use. The treated leaves were then decolored for 24 h in a chloral hydrate solution (5:2, w/v) and finally rinsed 3 times with ddH_2_O prior to being observed under an Olympus BX‐53 microscope.

### Sterile Culture

Wheat seeds of 8P4087 were washed with running water, surface sterilized with 70% ethanol for 1 min, and rinsed three times with sterile distilled water. They were soaked for 9 min in 3% sodium hypochlorite and 30 sec in 70% ethanol. After rinsing three times with sterile distilled water and air‐drying on sterile filter paper, they were cultured on MS medium with illumination for 16 h at 16°C for 21 d.

### DNA/RNA Extraction

Genomic DNA was extracted by using the cetyltrimethyl ammonium bromide (CTAB) method. Total RNA was extracted from leaves of normal bulks and autoimmunity bulks using the RNA simple Total RNA Kit (TianGen, China).

### Bulked Segregant RNA‐Sequencing

In the bulked segregant RNA‐Sequencing (BSR‐Seq) experiment, 30 homozygous necrotic and 30 homozygous normal F_2_ plants derived from the cross of 8P4087 × AK58 were selected randomly to compose the necrotic bulk and normal bulk for RNA‐Seq, respectively. Bulked RNA was sequenced on an Illumina HiSeq 4000 platform in the paired end mode of 150 bp at Beijing Novogene Bioinformatics Technology Co. Ltd, Beijing, China, following the manufacturer's standard protocol.^[^ ^46^ ^]^

### Development and Validation of Polymorphic Molecular Markers

Based on the BSR‐Seq result, the simple sequence repeat (SSR), single nucleotide polymorphism (SNP), and insertion/deletion (InDel) sequences that fell in the target genomic region of *WAI‐B2* in the Chinese Spring RefSeqv1.0 genome sequence (http://wheatomics.sdau.edu.cn/) were selected to design PCR primers using BatchPrimer3 v1.0. The designed SSR and InDel primers were screened for polymorphisms between the parental lines, as well as the necrotic and normal DNA bulks. After linked markers were identified, polymorphic markers were used to genotype the F_2_ individuals in the segregating populations. The genotypes and phenotypes of each recombinant were further confirmed in the F_3_ progenies for fine mapping *WAI‐B2*.

### Construction of Transgenic Vector

The genomic DNA fragment of *WAI‐B2*, including the 1,458 bp genomic region containing exons and introns, the 2,473 bp upstream putative native promoter sequence and the 1,260 bp downstream sequence, was cloned from 8P4087 and inserted into the pCAMBIA1300 vector to generate the complementary construct. The construct was transformed into Fielder via *Agrobacterium*‐mediated transformation.

### N. Benthamiana Cell Death and Subcellular Localization Assays

The full‐length coding sequences (CDS) of the *WAI‐B2* from 8P4087 and *wai‐B2* from ND399 were inserted downstream of the MAS promoter in the pS1300‐EGFP vector for *N*. *benthamiana* cell death assay (wai‐B2‐EGFP and WAI‐B2‐EGFP). After expression in *N*. *benthamiana* leaves via *Agrobacterium*‐mediated transformation, the HR‐like phenotype was observed 72 hpi after incubation at 22°C.

For subcellular localization assay, the *WAI‐B2* (p35S::WAI‐B2‐EGFP) and *wai‐B2* (p35S::wai‐B2‐EGFP) CDS sequences were inserted downstream of the 35S promoter in the pCAMBIA1300 vector. The WAI‐B2 was divided into two parts, the N‐terminus from 1–440 aa and the C‐terminus from 413‐451 aa, according to the transmembrane domain. Both parts included the transmembrane region from 413–440 aa of WAI‐B2. The two sequences were inserted downstream of the 35S promoter in the pCAMBIA1300 vector and fused with EGFP or mCherry and co‐expressed in *N*. *benthamiana* leaves via agroinfiltration. Subcellular localization assays were performed using Golgi marker (p35S::TaNBR1‐mCherry).^[^ ^47^ ^]^ Additionally, EGFP fusion constructs (p35S::WAI‐B2‐EGFP) and the mCherry construct (p35S::TaHsp70 and TaHsp90‐mCherry) were co‐expressed in *N*. *benthamiana* leaves by agroinfiltration. After 40 h of incubation at 22°C, GFP fluorescence and mCherry fluorescence of *N*. *benthamiana* leaves were observed using a laser confocal microscope.

### Phylogenetic Analysis

The CDS of WAI‐B2 was obtained from mutant 8P4087.The haplotype of the *gene* was analyzed using the sequenced genome, use BLAST analysis and constructed by using Geneious Prime.

The amino acid sequence of WAI‐B2 was obtained from mutant 8P4087. A BLAST analysis was conducted against the genome sequences of various species including common wheat, wild emmer wheat, durum wheat, the S genome group, *A. thaliana*, rice (*Oryza sativa*) and maize (*Zea mays*), using the WAI‐B2 amino acid sequence as a query. The top hit was regarded as homologs of WAI‐B2. The phylogenetic tree was constructed by using MEGA and Geneious Prime.

### Isolation and Transient Transfection of Wheat Protoplasts

The full‐length CDS of *WAI‐B2* and *wai‐B2* were inserted downstream of the maize ubiquitin promoter (*Ubi*) in the pJIT163UBI‐EGFP vector and pJIT163UBI‐mCherry vector, respectively. Isolation and transient transfection of leaf mesophyll cell protoplasts from wheat cultivar ND399 (two‐week‐old) were performed at room temperature. Each transfection experiment utilized 20 µg of plasmid DNA (1 µg µl^−1^). Buffer used in transfections is 1 m CaCl_2_, 1 m KOH, 2 m KCl, 1 m MgCl_2_ and 0.2 m MES (pH 5.7), stock solutions were prepared with double‐deionised water, filter sterilised and stored at 4 °C. On the day of transfection, 0.8 _M_ mannitol and working stock solutions W5 were freshly prepared. Use protocols for wheat protoplast isolation and transfection that are enabled by cellulase R‐10 and macerozyme R‐10 containing enzymatic solution and polyethylene glycol‐mediated method.^[^ ^48^ ^]^ Protoplasts were incubated in darkness for 16 h post‐transfection before imaging using a confocal laser scanning microscopy.

### Quantitative Reverse Transcription PCR (qRT‐PCR)

Total RNA was extracted from leaves of ND399, 8P4087, Fielder and three *WAI‐B2* transgenic lines at 6, 12, 18 and 24 DPG using the RNA simple Total RNA Kit (TianGen, China). qRT‐PCR analysis was conducted using the PrimeScript™ RT reagent Kit with gDNA Eraser (Perfect Real Time) (TaKaRa, Japan), TB Green Premix Ex Taq II (TaKaRa, Japan), with wheat β‐*TaActin* gene as the internal control. Each experiment included three biological replicates and three technical replicates. All bar graphs were drawn using GraphPad Prism v.8.0.2. Values of traits measured were expressed as the mean ±SD or SEM. The differences were statistically significant, as determined using a student's t‐test at *p* < 0.05, 0.01 or 0.001.

### Western Blotting

The full‐length CDS of *WAI‐B2* and *wai‐B2* were inserted downstream of the MAS promoter in the pS1300‐EGFP vector. After expression in *N*. *benthamiana* leaves via agroinfiltration, proteins were extracted from the leaves after 40 h (before cell death) of incubation at 22°C using the PROCAP GFP‐Magnetic IP/CoIP KIT (Lablead, China). The proteins were subjected to sodium dodecyl sulfate‐polyacrylamide gel electrophoresis (SDS‐PAGE) (Lablead, China) and resolved at 160 V for 35 min. Western blotting was performed and proteins were prepared for Liquid Chromatography (LC)‐Tandem Mass Spectrometry (MS/MS) analysis.

### Luciferase Complementation Imaging Assay

The luciferase complementation imaging (LCI) assays were conducted to assess the interaction between specified proteins in *N*. *benthamiana* leaves. The full‐length CDS of *TaHsp70* and *TaHsp90* were fused with the N‐terminal region of the firefly luciferase (nLuc) reporter gene (TaHsp70‐nLuc and TaHsp90‐nLuc), while *WAI‐B2* and *wai‐B2* were fused with the C‐terminal region of the firefly luciferase (cLuc) (cLuc‐WAI‐B2 and cLuc‐wai‐B2). These constructs were then transformed into *A. tumefaciens* strain GV3101. The CDS of these genes were amplified from ND399 using the primers listed in Table S3 (Supporting Information). Cells were resuspended in an infiltration buffer (10 mm MES, 10 mm MgCl_2_, and 200 µm acetosyringone, pH 5.7) at OD_600_ of 0.8, and infiltrated into three‐week‐old *N*. *benthamiana* leaves. The nLuc and cLuc derivative constructs were co‐infiltrated, and the luciferase activity was analyzed at 36 hpi using the Night SHADE LB 985N (Berthold, Germany). nLuc and cLuc fusion proteins were detected with StayBrite™ D‐Luciferin, Potassium Salt (Cat. No. 7903‐1G).

### Co‐IP Assay

The full‐length CDS of *TaHsp70* and *TaHsp90* were inserted into VC006‐pCambia1300‐221‐Myc vector (TaHsp90‐Myc and TaHsp70‐Myc) at the *Kpn*I/*Bam*HI sites, while the full CDS of *WAI‐B2* and *wai‐B2* was inserted into pS1300‐EGFP vector at th*e Xba*I*/Spe*I sites. After expression in *N*. *benthamiana* leaves via *Agrobacterium*‐mediated transformation, sampling was taken 40 h before incubation with 22 °C without HR‐like phenotype.

The total proteins were extracted and enrichment of WAI‐B2‐pS1300‐EGFP and wai‐B2‐pS1300‐GFP protein using PROCAP GFP‐Magnetic IP/CoIP KIT (PGM025), 10 µL sample was loaded for input immunoblot, and 15 µL sample was loaded for IP immunoblot. The proteins were detected using the primary antibodies of ProteinFind® Anti‐GFP Mouse Monoclonal Antibody (1:5000 dilution; TransGen Biotech, Catalog # HT801‐02), Ab32 Anti‐Myc tag antibody (1:5000 dilution; abcam, Catalog # Ab32) and the secondary antibody of Goat Anti‐Mouse IgG (H+L)‐HRP (1:3000; EarthOx, Catalog # E030110‐02). After development, it was washed with antibody stripping solution and then incubated with primary antibodies of Actin (Plant specific) Mouse mAb (1:5000 dilution; Abclonal, Catalog # AC009) and the secondary antibody of Goat Anti‐Mouse IgG (H+L)‐HRP (1:3000; EarthOx, Catalog # E030110‐02).

### Structure Prediction and WAI‐B2 Variants Design

SWISS‐MODEL (https://swissmodel.expasy.org/) was used to predict the protein structure of WAI‐B2 and its amino acid replacement proteins. The amino acids interaction with key amino acid sites and their adjacent amino acids were analyzed by PyMOL (https://pymol.org/). New variants on the L425 site were designed to change its interacting distance and site and tested in *N*. *benthamiana* leaves. Leucine at position 425 was replaced with tryptophan (W) and tyrosine (Y) (referred to as WAI‐B2^L425W^ and WAI‐B2^L425Y^), which are also aromatic amino acids, respectively. Then the amino acids were divided into four groups according to their physical and chemical properties, and one of each was selected as a representative for substitution. Aspartic acid (D) was selected among the acidic amino acids, Histidine (H) was selected among the basic amino acids, Asparagine (N) was selected among the neutral amino acids, and Valine (V) was selected among the non‐polar hydrophobic amino acids.

## Conflict of Interest

The authors declare no conflict of interest.

## Author Contributions

W.L. and Y.C. contributed equally to this work. Q.W., Z.L., H.J. and Y.Z. conceived the study and designed the experiments. L.D., G.G., H.Z., G.W., L.D., P.L., M.L., D.Q., K.Z., B.L., Y.H., X.C., B.H., F.Y., H.F., Z.L., J.H. and H.L. provided valuable advice for the experimental design, results interpretation and manuscript modification. H.Z., T.S., S.C., Z.L., L.K., and W.Y. performed the assessment of disease resistance. G.G., D.L., and Y.Q. performed bioinformatic analysis. Q.W., Z.L., H.J., Y.Z., and H.L. revised the manuscript. W.L., and Y.C. wrote the manuscript. All authors contributed to the article and approved the manuscript.

## Supporting information



Supporting Information

## Data Availability

The data that support the findings of this study are available in the supplementary material of this article.
