# Supplementary material for: A Transmembrane Protein WAI‐B2 Confers Multiple Disease Resistance in Wheat by Activating Autoimmunity
Source: Adv Sci (Weinh). 2025 Oct 28;13(3):e11576. doi: 10.1002/advs.202511576 (PMC12806210; doi:10.1002/advs.202511576)
Supplement: Supplementary file 1 — Supporting Information [file ADVS-13-e11576-s001.docx]

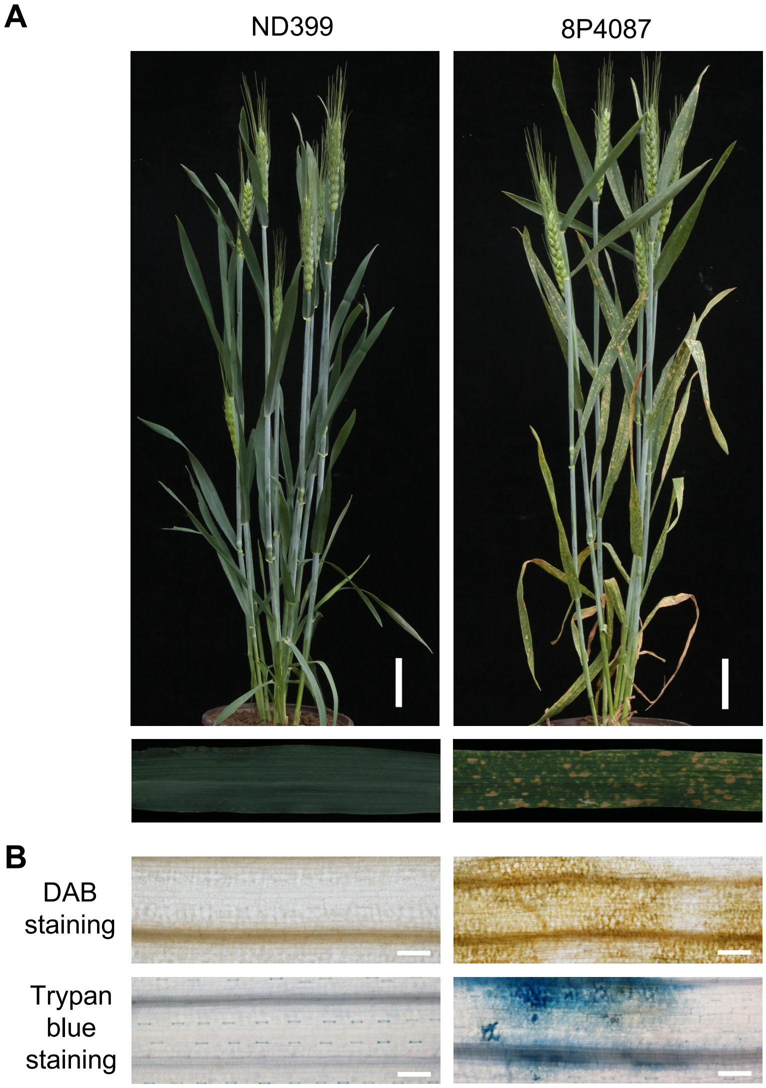

Figure S1. Phenotypic and physiological characterizations of the wild-type ND399 and the WAI mutant 8P4087. A) Morphologies of ND399 and 8P4087. B) DAB and Trypan blue staining of ND399 and 8P4087 leaves at spontaneous leaf spot starting stage. Scale bars: 5 cm (A); 200 μm (B).

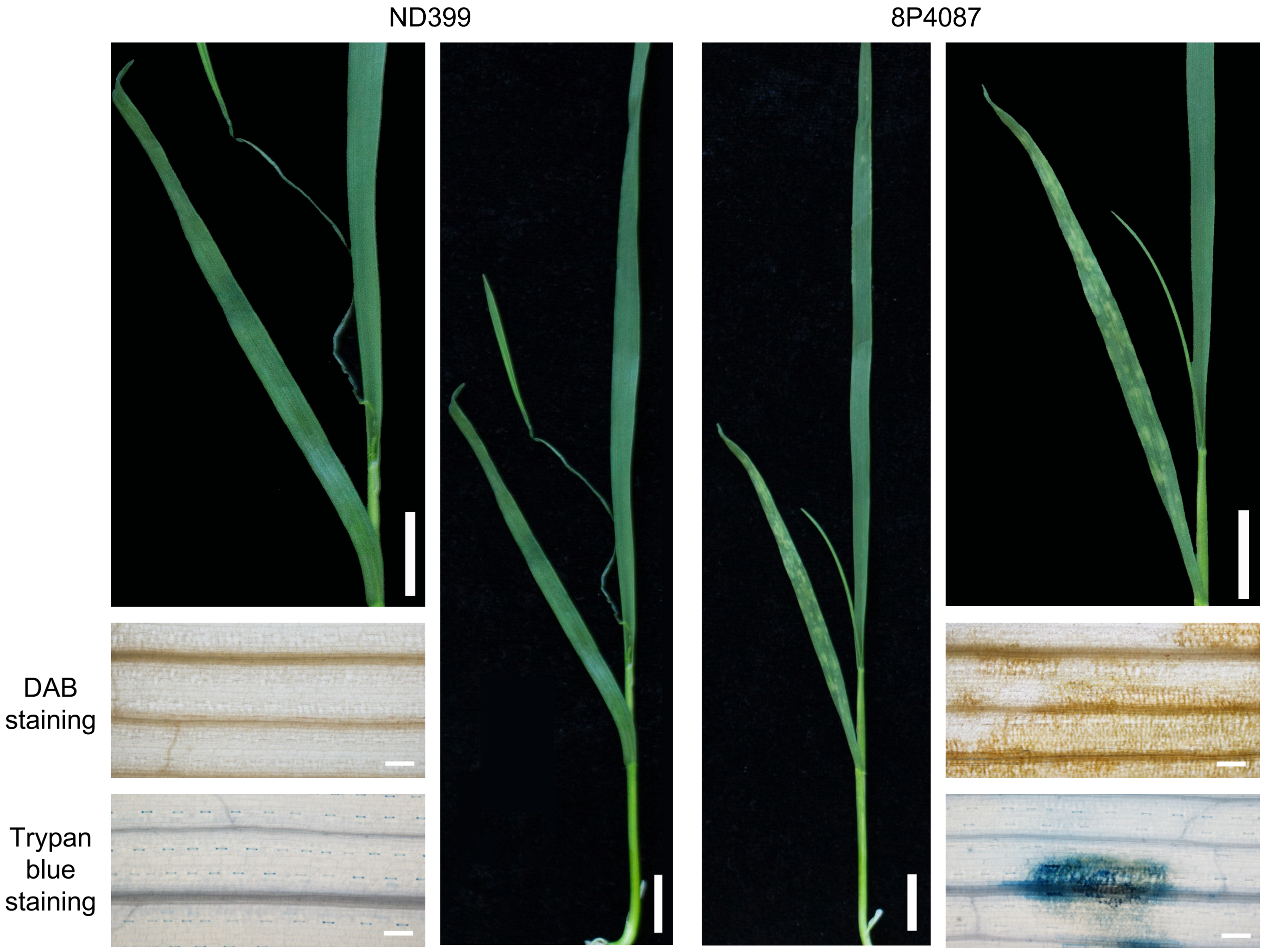

Figure S2. Phenotypic and physiological characterizations of the wild-type ND399 (left) and the WAI mutant 8P4087 (right) under sterile growth condition. ND399 and 8P4087 were grown in MS culture medium under sterile condition. Scale bars: 1 cm in phenotypic characterization; 200 μm in DAB and Trypan blue staining.

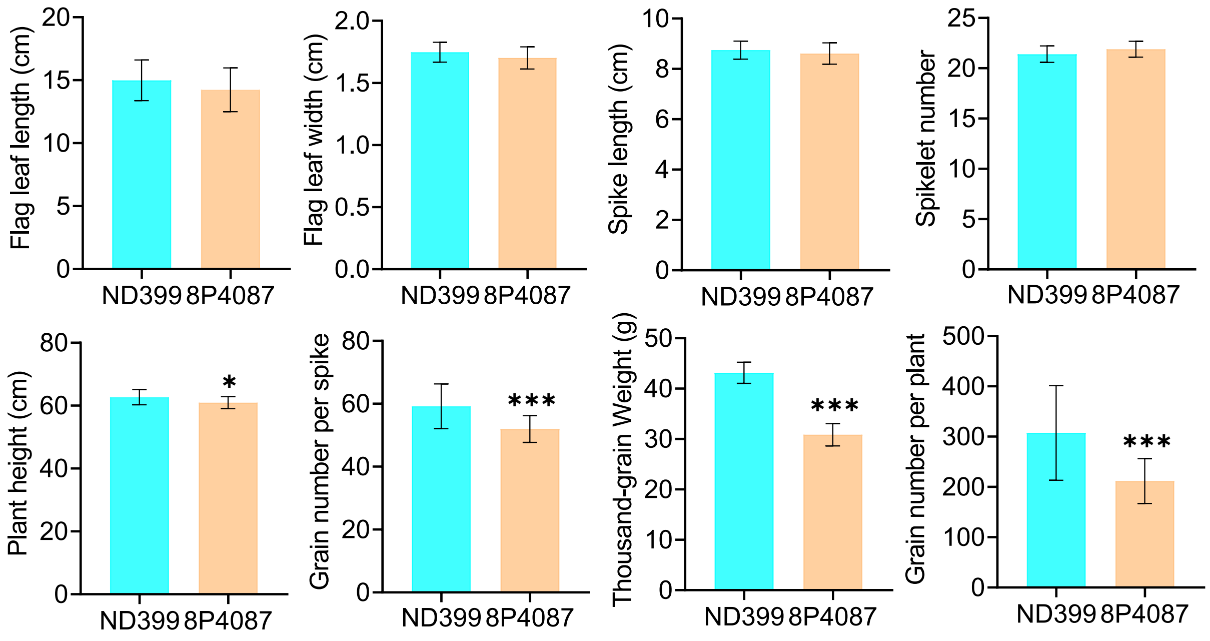

Figure S3. Statistical analysis of main agronomic traits of ND399 and 8P4087. Data are presented as means ± SD (*n* = *20*). Twenty independent plants were measured to calculate the means value. Statistically significant differences at *$p < 0.05$, **$p < 0.01$ or ***$p < 0.001$ were detected using *t*-tests.

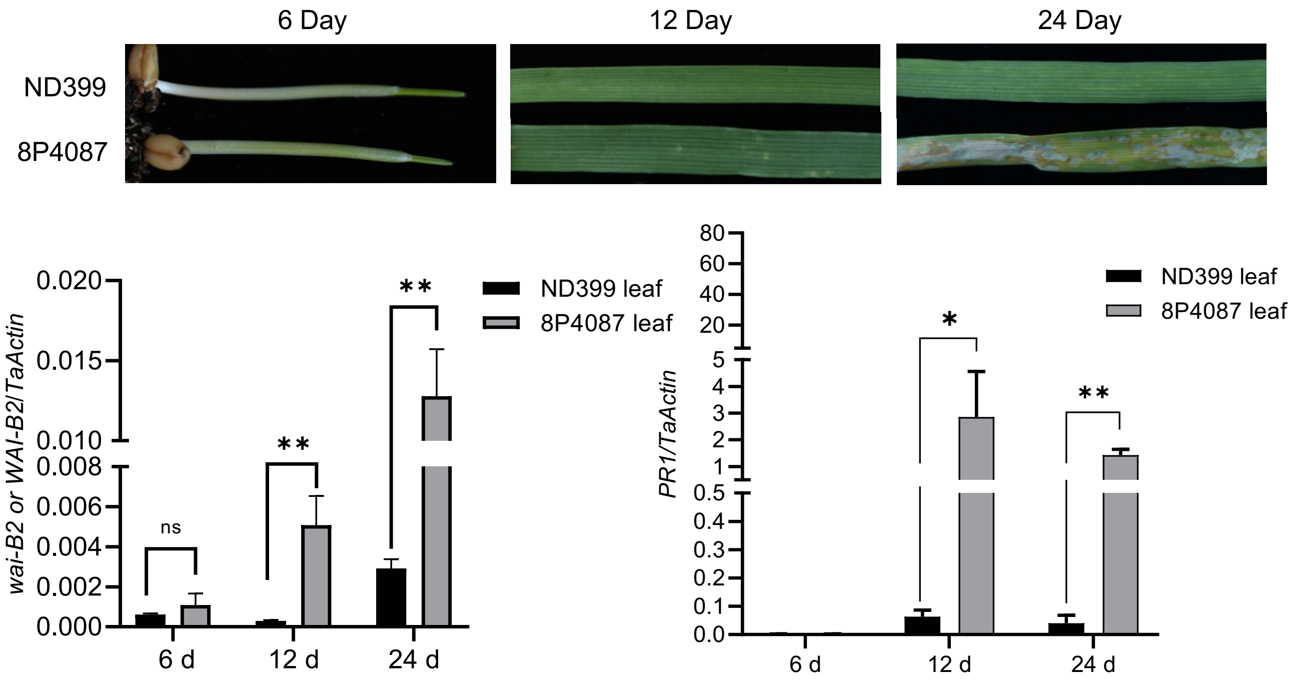

Figure S4. The expression pattern of *WAI-B2* and *wai-B2*. Morphologies of ND399 and mutant 8P4087, and the expression level of *wai-B2*/*WAI-B2* and *PR1* genes in the leaves of ND399 and 8P4087 plants grown at 16° C in the growth chamber. Values are the means ± SD, $n = 3$ biologically independent samples. Three independent plants are measured to calculate the means value. Statistically significant differences at $*p < 0.05$, $**p < 0.01$ or $***p < 0.001$ were detected using *t*-tests.

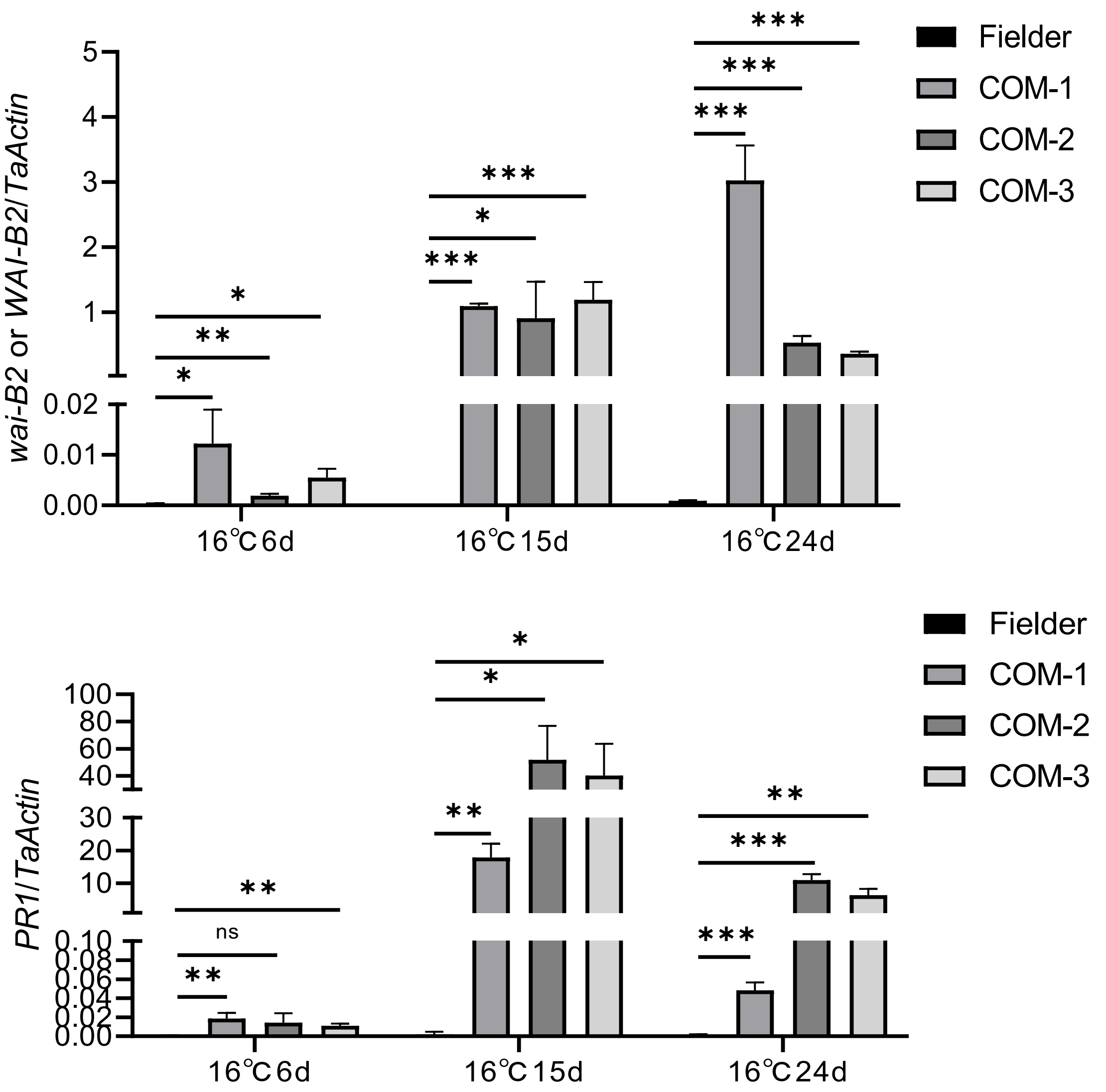

Figure S5. The expression pattern of *WAI-B2* and *PR1*. The expression level of *WAI-B2* and *PR1* genes in the leaves of the transgenic positive plants COM-1, COM-2, and COM-3 grown at 16° C in the growth chamber. Values are the means ± SD, $n = 3$ biologically independent samples. Three independent plants are measured to calculate the means value. Statistically significant differences at *$p < 0.05$, **$p < 0.01$ or ***$p < 0.001$ were detected using *t*-tests.

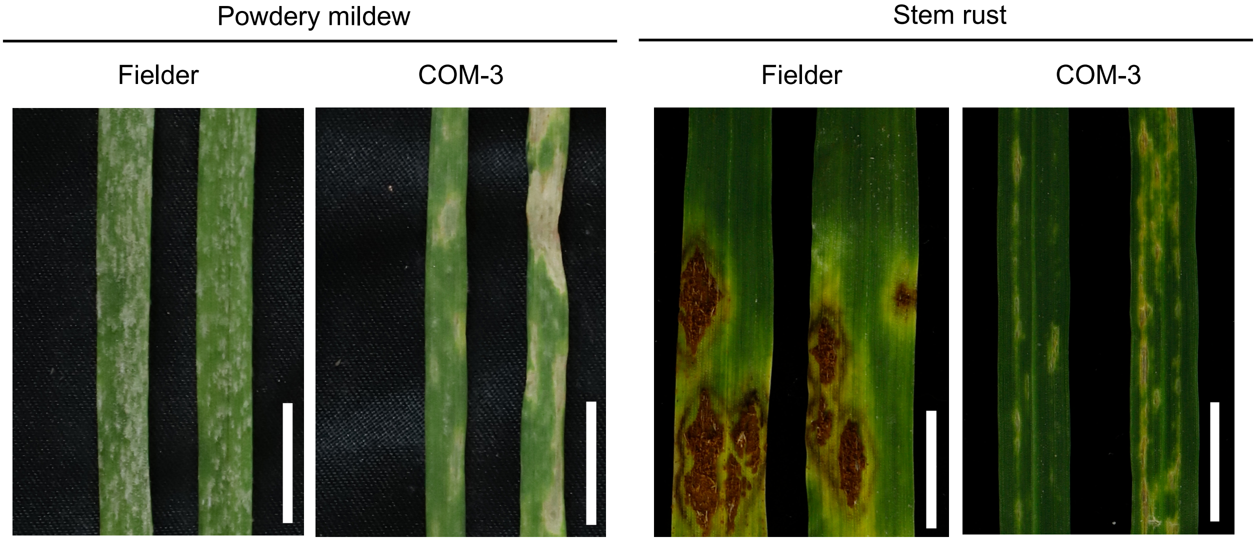

Figure S6. Reactions of Fielder and *WAI-B2* transgenic line COM-3 to powdery mildew and stem rust at the seedling stage. Scale bars: 1 cm.

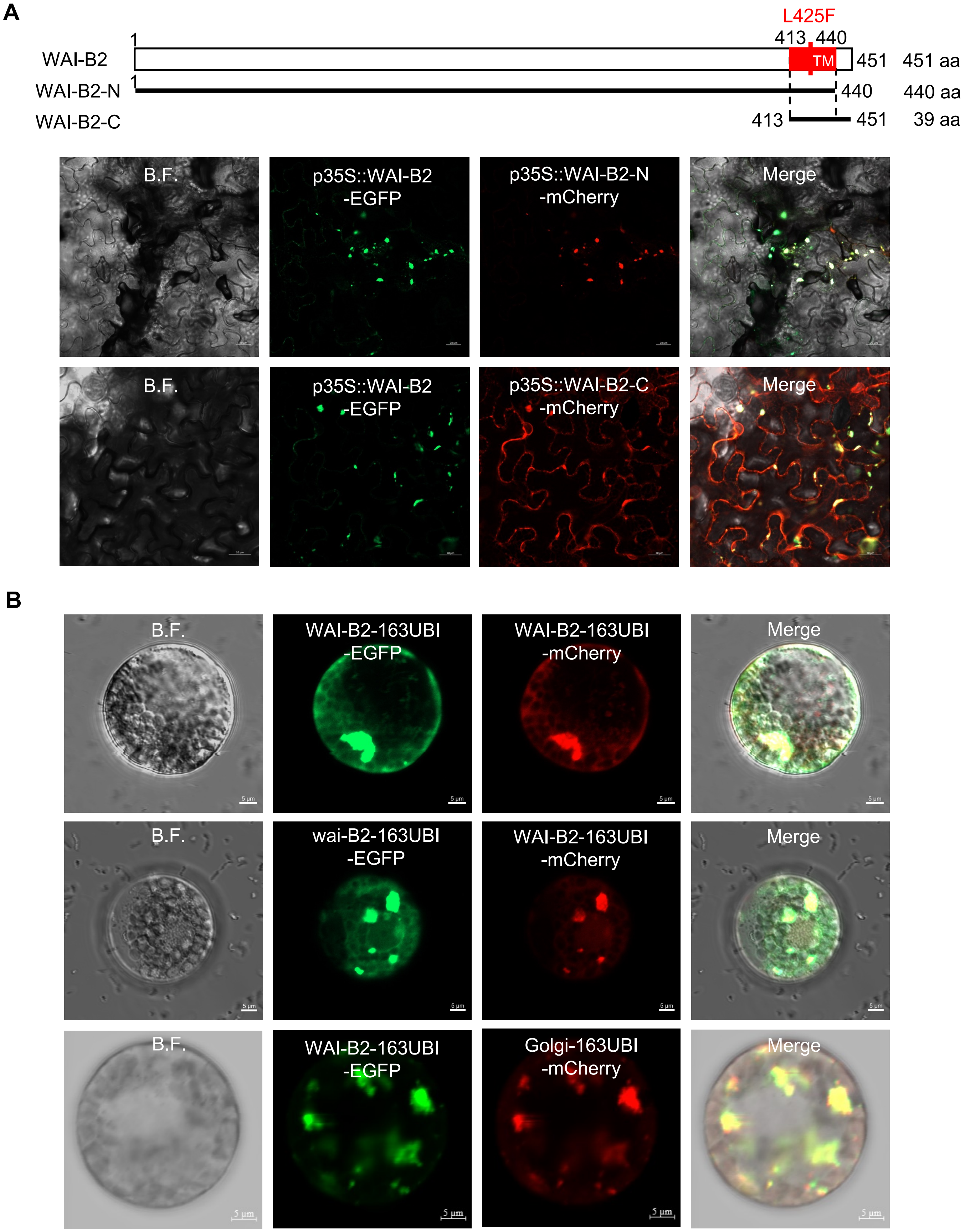

Figure S7. Subcellular localization of WAI-B2. A) Subcellular localization of p35S::WAI-B2-N-mCherry (top) and p35S::WAI-B2-C-mCherry (bottom) with p35S::WAI-B2-EGFP in *N. benthamiana*. Scale bars: 20 μm. B) Subcellular localization of WAI-B2 and wai-B2 in wheat protoplasts. WAI-B2-163UBI-mCherry was transfected into wheat leaf mesophyll protoplasts by the PEG-mediated method with a WAI-B2-163UBI-EGFP (top) and a wai-B2-163UBI-EGFP (center), respectively. WAI-B2-163UBI-EGFP was transfected with a Golgi-163UBI-mCherry (bottom). Scale bars: 5 μm.

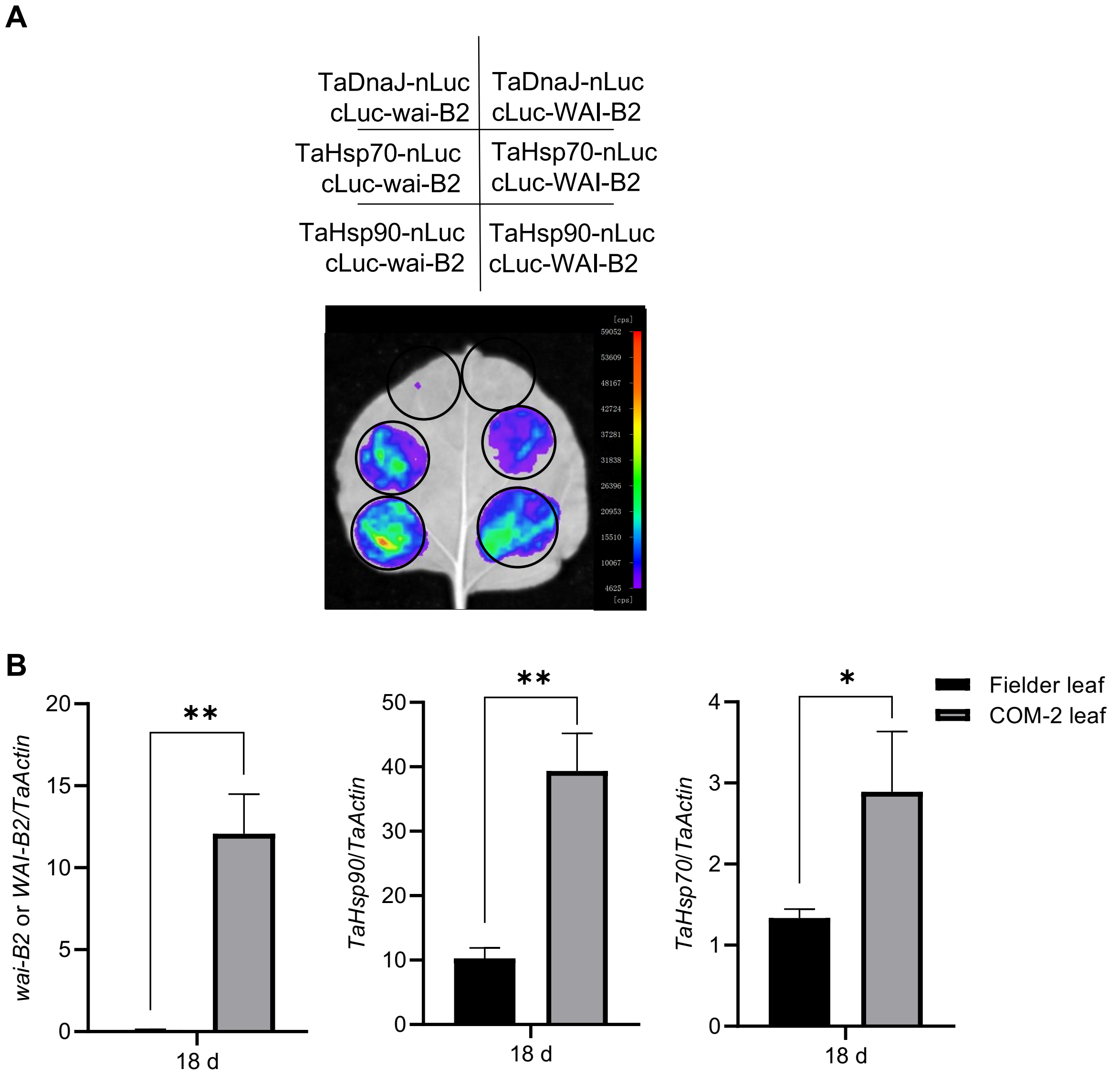

Figure S8. WAI-B2 interacts with the TaHsp90 and TaHsp70. A) LCI assay of cLuc-WAI-B2 and cLuc-wai-B2 with TaHsp90-nLuc, TaHsp70-nLuc and TaDnaJ-nLuc in *N. benthamiana* leaves. The pseudo-color bar indicates the range of luminescence intensity. B) The expression level of *wai-B2*/*WAI-B2*, *TaHsp90* and *TaHsp70* genes in the leaves of Fielder and transgenic plant COM-2 by qPCR. Values are the means ± SD, n = 3 biologically independent samples. Three independent plants are measured to calculate the means value. Statistically significant differences at $*p < 0.05$, $**p < 0.01$ or $***p < 0.001$ were detected using *t*-tests.

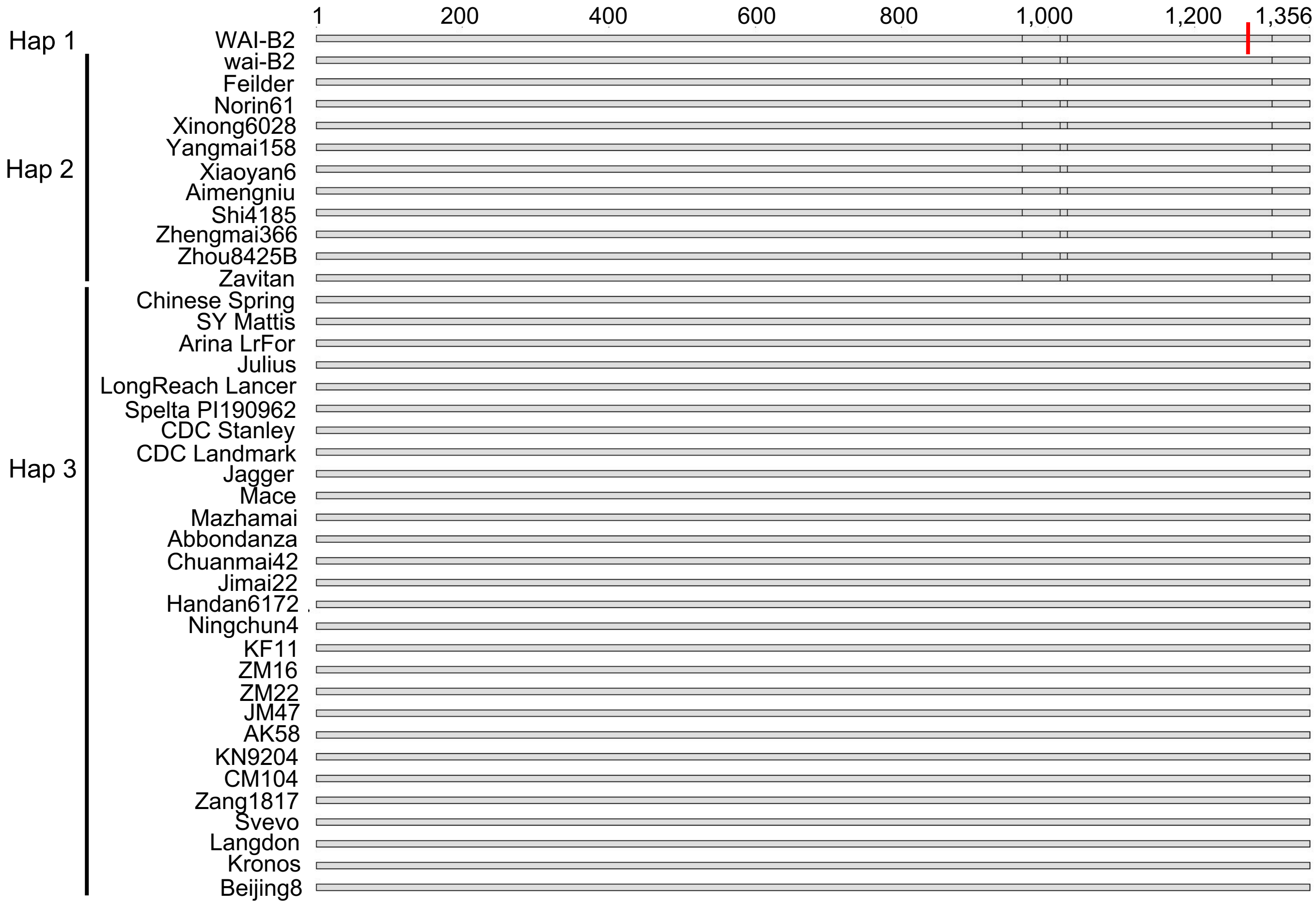

Figure S9. Haplotype analysis of *wai-B2* and *WAI-B2* in the hexaploid and tetraploid wheat reported on the WheatOmics 1.0 (http://202.194.139.32/). Black short vertical lines indicated SNP. The SNP in the red bar was *WAI-B2* specific mutation sites.

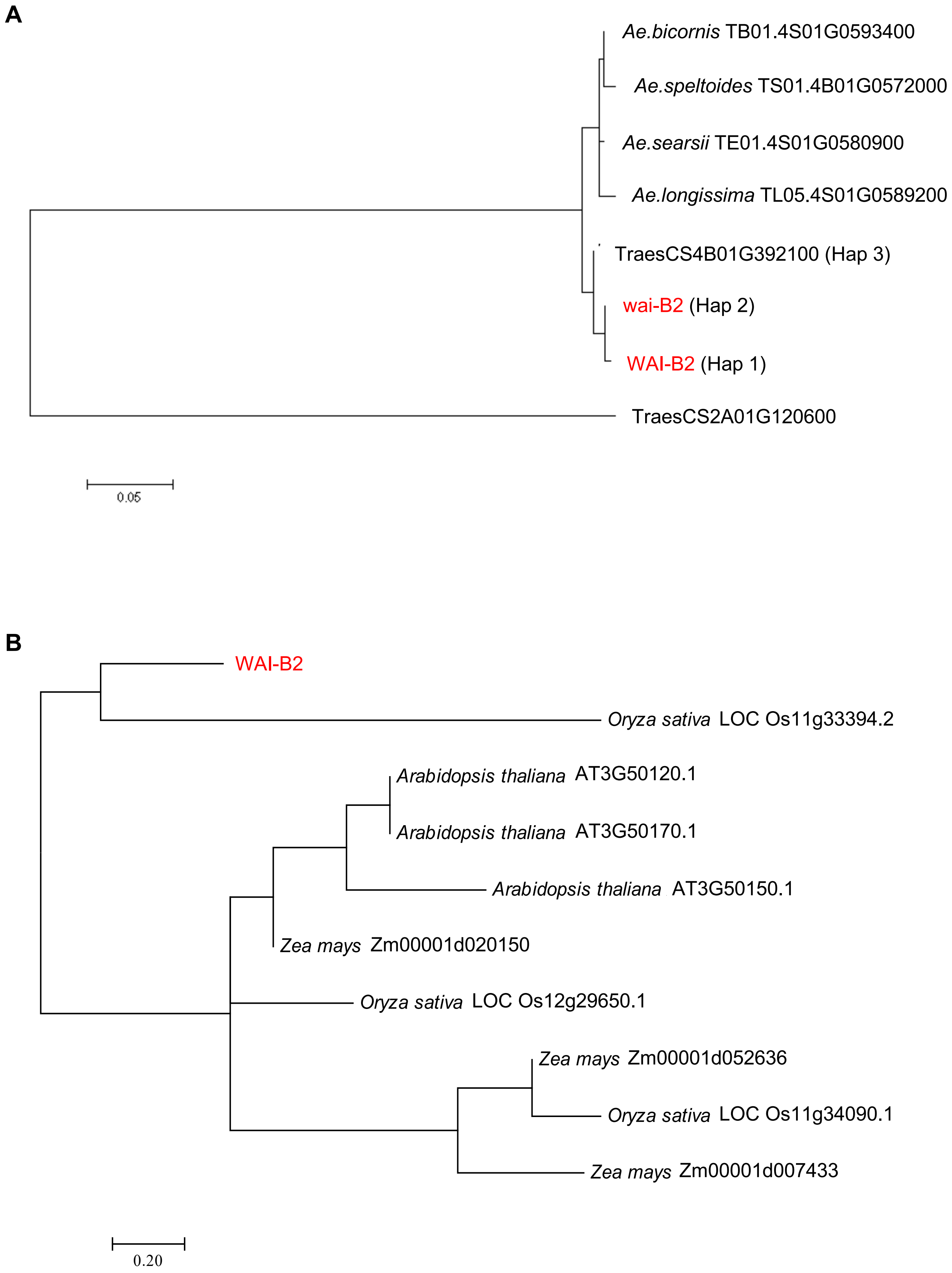

Figure S10. Phylogenetic tree of WAI-B2 and homologous proteins. A) Cluster analysis of WAI-B2 homologous proteins in published *Triticeae* genomes, *Ae. speltoides*, *Ae. longissimi*, *Ae. bicornis*, *Ae. searsii, protein TraesCS2A01G120600 was used as controls. B) C*luster analysis of WAI-B2 homologous proteins in plant species, including *Arabidopsis thaliana, Zea mays* and *Oryza sativa.*
